# Sesterterpenoid and Steroid Metabolites from a Deep-Water Alaska Sponge Inhibit Wnt/β-Catenin Signaling in Colon Cancer Cells

**DOI:** 10.3390/md16090297

**Published:** 2018-08-27

**Authors:** Hyun Bong Park, Nguyen Quoc Tuan, Joonseok Oh, Younglim Son, Mark T. Hamann, Robert Stone, Michelle Kelly, Sangtaek Oh, MinKyun Na

**Affiliations:** 1 Department of Chemistry, Yale University, New Haven, CT 06520, USA; hyunbong.park@yale.edu (H.B.P.); joonseok.oh@yale.edu (J.O.); 2 Chemical Biology Institute, Yale University, West Haven, CT 06516, USA; 3 Department of Pharmacognosy, College of Pharmacy, Chungnam National University, Daejeon 34134, Korea; quoctuan3012@naver.com; 4 Phúthọ College of Pharmacy, Viettri City, Phúthọ Province 293500, Vietnam; 5 Department of Bio and Fermentation Convergence Technology, BK21 PLUS program, Kookmin University, Seoul 136-702, Korea; wwt245@naver.com; 6 Department of Drug Discovery and Biomedical Sciences, Medical University of South Carolina, Charleston, SC 29425, USA; hamannm@musc.edu; 7 Auke Bay Laboratories, Alaska Fisheries Science Center, NOAA National Marine Fisheries Service, Juneau, AK 99801, USA; bob.stone@noaa.gov; 8 Coast and Oceans National Centre, National Institute of Water and Atmospheric Research, Auckland Central 1149, New Zealand; Michelle.Kelly@niwa.co.nz

**Keywords:** natural products, marine sponge, sesterterpenoid, steroid, colorectal cancer, Wnt, β-catenin, Alaska

## Abstract

The Wnt/β-catenin signaling pathway is known to play critical roles in a wide range of cellular processes: cell proliferation, differentiation, migration and embryonic development. Importantly, dysregulation of this pathway is tightly associated with pathogenesis in most human cancers. Therefore, the Wnt/β-catenin pathway has emerged as a promising target in anticancer drug screening programs. In the present study, we have isolated three previously unreported metabolites from an undescribed sponge, a species of *Monanchora* (Order Poecilosclerida, Family *Crambidae*), closely related to the northeastern Pacific species *Monanchora pulchra*, collected from deep waters off the Aleutian Islands of Alaska. Through an assortment of NMR, MS, ECD, computational chemical shifts calculation, and DP4, chemical structures of these metabolites have been characterized as spirocyclic ring-containing sesterterpenoid (**1**) and cholestane-type steroidal analogues (**2** and **3**). These compounds exhibited the inhibition of β-catenin response transcription (CRT) through the promotion of β-catenin degradation, which was in part implicated in the antiproliferative activity against two CRT-positive colon cancer cell lines.

## 1. Introduction

Colorectal cancer is the fourth most prevalent cancer and the third leading cause of cancer-related mortality worldwide. In 2017, approximately 130,000 new cases and 50,000 deaths can be attributed to colorectal cancer in the United States alone [1]. Despite significant advances in surgery, radiotherapy and chemotherapy, the survival rate of colorectal cancer patients has only slightly increased over the past decade [1]. Besides, emerging side effects and drug resistances are likely unavoidable, which in turn counteract current chemotherapeutic approaches in the cancer treatment. Therefore, extensive investigations of potential chemotypes along with mechanisms of action are significantly required for the development of effective anticancer drug lead structures.

The Wnt/β-catenin signaling cascade is one of the well-established cell intrinsic canonical pathways involved in a variety of biological events [2]. A multitude of research has also shown that abnormal activation of the Wnt/β-catenin pathway frequently occurs in various cancer types (e.g., colorectal, leukemia, prostate and breast) [3]. Subsequently, the functional loss of a tumor suppressor known as adenomatous polyposis coli (APC) was found to be highly associated with the aberrant activation of the oncogenic pathway [3]. The APC was initially identified as the main cause of familial adenomatous polyposis (FAP) syndrome; thereafter it was reported that the genes encoding APC are commonly mutated in most sporadic colorectal cancers [4,5]. The interplay between APC and β-catenin, a central transcriptional regulator, has been well elucidated in the pathway. The mutational alteration of the APC gene stimulates the stabilization of β-catenin that abnormally accumulates and moves to the nucleus in order to bind to T-cell factor/lymphocyte enhancer factor (Tcf/LEF) [6]. The β-catenin/Tcf/LEF transcription ultimately regulates the expression of Wnt/β-catenin target genes such as c-Myc and cyclin D1, thereby culminating in tumorigenic processes in the cells [7,8]. A body of evidence has made it apparent that inhibition of the oncogenic β-catenin pathway significantly suppresses the carcinogenesis of cancers [9,10]. Hence, discovering effective inhibitors targeting β-catenin stabilization and downstream signaling could be of high importance.

Previously, we have reported that the flavonoid galangin effectively inhibits β-catenin response transcription (CRT) by promoting β-catenin degradation in colon cancer cell lines [11], and new 4,9-friedodrimane-type sesquiterpenoids, isolated from a marine sponge, suppress β-catenin expression and exhibit cytotoxic activity in colon cancer cells [12]. Emerging evidence indicates that marine sponges are a prolific source of biologically active natural products [13,14]. Extensive chemical studies with marine sponges have identified unique and diverse structural characteristics, many of which are functionally characterized in a wide range of promising pharmaceutical relevance [15,16,17]. In particular, marine sponge species belonging to the genus *Monanchora* (order Poecilosclerida, family *Crambeidae*) have yielded a variety of new natural products. Examples include a unique family of polycyclic guanidine alkaloids exhibiting diverse bioactivities, including cytotoxicity, antibiotic, antiviral, and anti-inflammatory [18,19,20,21,22,23]. Cytotoxic sesterterpenoids and anti-inflammatory steroids have been isolated from a *Monanchora* sp., collected in Korean waters [24,25]. A number of sterols have also been elucidated from this genus [26,27]. Based on these diverse findings, we herein report the investigation of an undescribed species of *Monanchora*, closely related to the Northeastern Pacific species, *Monanchora pulchra* [28], collected from deep waters off the Aleutian Islands of Alaska. The details include structural characterization of **1**–**3** via spectroscopic and spectrometric approaches, and quantum mechanics-based chemical shifts calculation with support of the advanced statistics DP4 [29]. The isolated compounds were also evaluated for their inhibitory activity of β-catenin response transcription (CRT) and antiproliferative activity against CRT-positive colon cancer cells.

## 2. Results and Discussion

**Figure 1:**
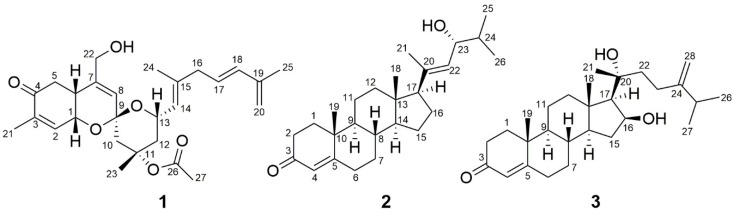
Chemical structures of **1**–**3.**

The fresh sponge (~1 kg) was immediately frozen on site and stored at −30 °C. The material was subsequently thawed, cut into small pieces and extracted with methanol to yield a crude methanol extract (53.2 g). The crude extract (2.5 g) was then subjected to reversed-phase C_18_ medium-pressure liquid chromatography (MPLC), followed by HPLC purification, yielding **1** (3 mg), **2** (3 mg) and **3** (2 mg) (Figure 1).

**Figure 2:**
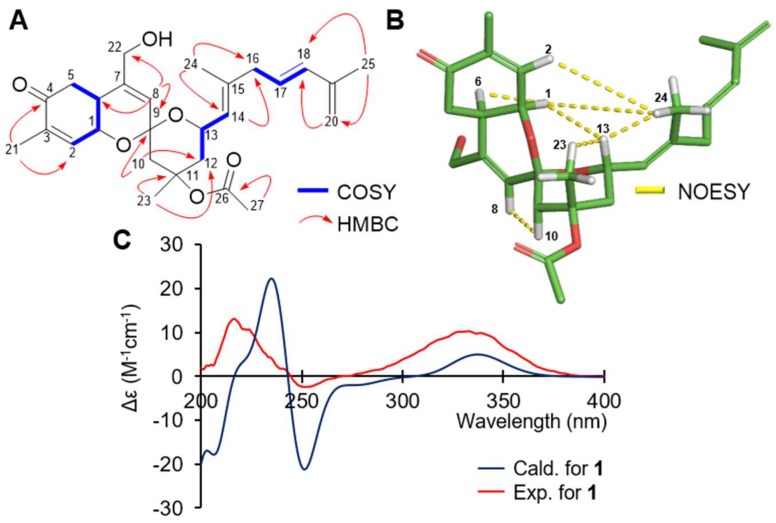
(**A**) Key COSY and HMBC correlations, (**B**) Key NOESY correlations, (**C**) Overlay of calculated (B3LYP/6-31G(d,p)) and experimental ECD spectra of **1**.

The molecular formula of **1** was deduced to be C_27_H_36_O_6_ based on HRESIMS data (obsd. [M + Na]^+^ *m*/*z* 479.2407, calcd. 479.2410) (Supplementary Materials, Figure S1). The chemical structure of **1** was established by the integrated analyses of 1D (^1^H and ^13^C) and 2D NMR (COSY, HSQC, HMBC, and NOESY) spectra (Table 1 and Supplementary Materials, Figures S2–S7). In brief, HMBC correlations from a singlet of a vinyl methyl (H_3_-21, *δ*_H_ 1.86) to a ketocarbonyl carbon (C-4, *δ*_C_ 198.8) and an olefinic carbon (C-2, *δ*_C_ 139.4) suggested the presence of a methyl-substituted α,β-unsaturated carbonyl moiety. Consecutive COSY cross-peaks from H-2 (*δ*_H_ 6.62) to H_2_-5 (*δ*_H_ 2.59 and 2.49) permitted the construction of a 2-methyl-2-cyclohexenone functionality (Figure 2A).

The HMBC cross peaks from H-5 and an oxygenated methine (H-1, *δ*_H_ 4.47) to a deshielded quaternary carbon (C-7, *δ*_C_ 142.5), along with the observation of an additional olefinic proton resonance at *δ*_H_ 5.66 (H-8), rendered the further structure of the aforementioned-constructed ring to be a bicyclic *δ*-lactone core of 2*H*-chromene (Figure 2A). The connectivity of an oxygen-bearing methylene group at *δ*_H_ 4.12 (H_2_-22) to C-7 was evident by the analysis of the HMBC spectrum, revealing the three-bond correlation from H-8 (*δ*_H_ 5.66) to C-22 (*δ*_C_ 63.8) (Figure 2A). Further analysis of 1D and 2D NMR spectra revealed the existence of a vinyl methyl singlet at *δ*_H_ 1.78 (H-23), a proton of the oxymethine carbon at *δ*_H_ 4.65 (H-13), and a highly oxygenated secondary carbon resonance at *δ*_C_ 96.8, suggesting the presence of a spiroketal-containing tricyclic ring system. This was confirmed by the HMBC cross-peaks from H-8 and diastereotopic methylene protons at *δ*_H_ 2.32 and 1.87 (H_2_-10) to the spiroketal carbon at *δ*_C_ 96.8 (C-9) (Figure 2A). The unusual tricyclic architecture of **1** is similar to those of the reported marine sponge-derived sesterterpenoids phorbaketals and alotaketals [30,31]. Indeed, an exomethylene (*δ*_H_ 4.90, *δ*_C_ 115.3) and an acetoxy group (*δ*_H_ 1.96, *δ*_C_ 22.6) validated by the presence of the ester carbonyl (*δ*_C_ 170.3) indicated that the structure of **1** is structurally closer to alotaketal C [32]. Additionally, the ^1^H NMR spectrum of **1** revealed the (*E*) C17-C18 olefinic geometry based upon the coupling constant (*J* = 15.6 Hz) between the two olefinic protons (*δ*_H_ 5.61 and 6.15) (Figure 2). Three-bond HMBC cross-peaks from a terminal methyl at *δ*_H_ 1.84 (H_3_-25) and exocyclic methylene protons at *δ*_H_ 4.90 (H_2_-20) to C-18 (*δ*_C_ 135.2) confirmed the unsaturation between C-17 and C-18 (Figure 2A).

The relative configuration of **1** was established by an analysis of the NOESY spectrum (Figure 2B). A strong NOESY correlation between H-1 (*δ*_H_ 4.47) and H-6 (*δ*_H_ 2.54) suggested that these protons were cofacial, establishing the *cis*-fused ring system. NOESY correlations from H-8 to H-10 and, from H-1 to H-13, supported the presence of the tricyclic moiety possessing a 9S* spiroketal junction (Figure 2B). NOESY cross-peaks between a vinyl methyl at *δ*_H_ 1.74 (H_3_-24) and H-2/H-13 established the (*Z*)-geometry of C14/15. Meanwhile, additional NOESY correlation from H-13 to H_3_-23 was observed, allowing they are on the same face, which is distinct from alotaketal C [32].

Assignment of the absolute configuration of **1** was achieved by comparison of its experimental and calculated electronic circular dichroism (ECD) spectra (Supplementary Materials, Figure S8 and Table S1). The experimental ECD spectrum of **1** revealed strong positive Cotton effects at ~212 and 330 nm, and a negative Cotton effect at ~250 nm, which was consistent with that of a simulated ECD spectrum of **1** (Figure 2C).

Only a few tricyclic spiroketal framework-containing sesterterpenoids have been characterized from several marine sponges. To the best of our knowledge, the first documented examples are alotaketals from the marine sponge *Hamigera* sp. and phorbaketals, isolated from the marine sponge *Phorbas* sp. [30,31]. Thereafter, the chemical structures of their derivatives have been reported from other sponge species [33,34,35]. These molecules revealed a myriad of biological properties such as inhibition of adipocyte differentiation, mast cell differentiation activity, cytotoxicity, and anti-inflammatory [31,36,37]. Aloketals have also been shown to induce potent activation of the cyclic adenosine monophosphate (cAMP) signaling pathway which is relevant to a number of human diseases [30].

**Figure 3:**
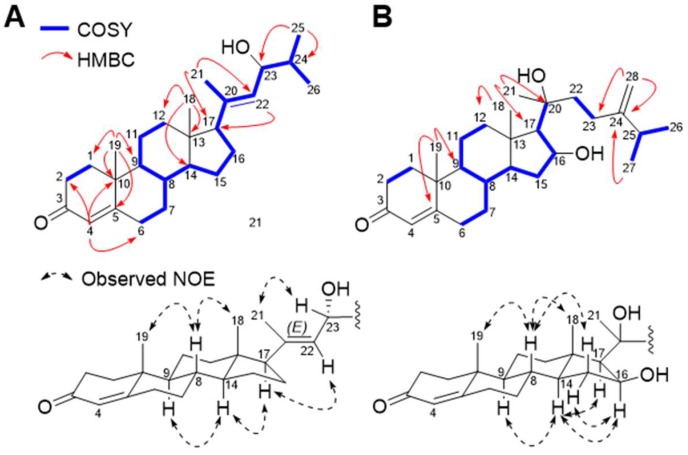
Key COSY, HMBC and NOE correlations of **2** (**A**) and **3** (**B**).

The molecular formula of **2** was determined as C_26_H_40_O_2_ (obsd. [M + Na]^+^ *m*/*z* 407.2926, calcd. 407.2926) by the analysis of HRESIMS data (Supplementary Materials, Figure S9), requiring seven degrees of unsaturation. The ^1^H NMR spectrum of **2** showed two olefinic protons ((*δ*_H_ 5.73, s) and (*δ*_H_ 5.24, d, *J* = 8.8 Hz)), one oxygen-bearing proton (*δ*_H_ 4.15, dd, *J* = 8.8, 6.7 Hz), and five methyl resonances ((*δ*_H_ 0.63, s), (*δ*_H_ 0.88, d, *J* = 6.7 Hz), (*δ*_H_ 0.96, d, *J* = 6.7 Hz), (*δ*_H_ 1.18, s), and (*δ*_H_ 1.70, s)) (Table 2 and Supplementary Materials, Figure S10). The ^13^C and HSQC NMR spectra revealed 26 well-resolved carbon resonances, including a ketocarbonyl (*δ*_C_ 199.8), a highly deshielded olefinic quaternary carbon (*δ*_C_ 171.6), two protonated olefinic carbons (*δ*_C_ 124.0 and 128.1), one olefinic quaternary carbon (*δ*_C_ 138.5), and an oxygenated methine carbon (*δ*_C_ 73.7). This is reminiscent of a framework of norcholestane (Supplementary Materials, Figures S11 and S13). The observation of the olefinic proton singlet at *δ*_H_ 5.73 and a ketocarbonyl carbon at *δ*_C_ 199.8 indicated the presence of an α,β-conjugated carbonyl group in the A-ring, which was supported by the HMBC cross-peaks from H_2_-1 (*δ*_H_ 2.02 and 1.67) and H-4 (*δ*_H_ 5.73) to C-3 (*δ*_C_ 199.8) (Figure 3A). The ^1^H-^1^H COSY cross-peaks starting from two doublets of the two methyl resonances at *δ*_H_ 0.96 (d, *J* = 6.7 Hz, H_3_-25) and at *δ*_H_ 0.88 (d, *J* = 6.7 Hz, H_3_-26) to H-17 (*δ*_H_ 2.08) constructed the steroidal side chain of 2,5-dimethylhex-4-en-3-ol. This is confirmed by HMBC cross-peaks from H_3_-21 (*δ*_H_ 1.70) to C-17 (*δ*_C_ 59.1) and C-22 (*δ*_C_ 128.1), and from H_3_-25 and H_3_-26 to C-23 (*δ*_C_ 73.7) (Figure 3A).

The relative configuration of **2** was established by NOESY spectral analysis. The NOESY cross-peaks between two methyls (Me-18; *δ*_H_ 0.63, s) and (Me-19; *δ*_H_ 1.18, s) and H-8 (*δ*_H_ 1.52, m), and between H-9 (*δ*_H_ 0.94, m)/H-14 (*δ*_H_ 1.08, m) and H-17 (*δ*_H_ 2.08, t, *J* = 9.5 Hz) were indicative of the same relative configuration as in naturally-occurring steroidal core structures (Figure 3A). The geometry of double bond between C-20 and C-22 was established as *E* by NOESY correlations between H-21/H-23, and H-17/H-22 (Figure 3A).

**Figure 4:**
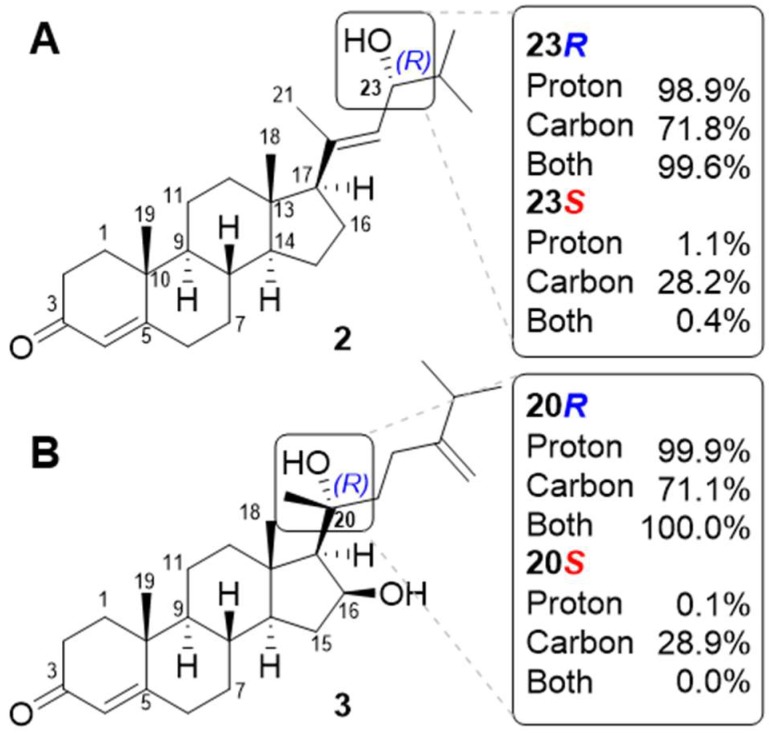
DP4 probabilities for **2** (**A**) and **3** (**B**).

The assignment of relative configuration of C-23 was achieved by the gauge-including atomic orbital (GIAO) NMR chemical shifts calculation. Conformational searches of two possible diastereomers a (23*R*) and b (23*S*) of **2** were conducted utilizing Macromodel (Schrödinger LLC) with a relative energy level in the MMFF94 force field, yielding 20 and 22 conformers within 10 kJ/mol, respectively (Supplementary Materials, Table S2). The chemical shifts of those conformers were subsequently computed at the B3LYP/6-31G(d,p) theory level using the Gaussian 09 package (Gaussian Inc.) and the resultant NMR properties were Boltzmann-averaged at 298.15 K (Supplementary Materials, Table S2). The ^1^H and ^13^C NMR chemical shift values of the diastereomers a and b were compared with those of the experimental NMR data of **2** (Supplementary Materials, Table S3) using the advanced statistics DP4 [29]. The statistical analyses revealed that the probability of diastereomer a (23*R*) is 99.6% in the consideration of both ^1^H and ^13^C NMR chemical shift values (Figure 4A, Supplementary Materials, Figure S16), establishing the relative configuration of C-23 of **2** as 23R*. Thus, the chemical structure of **2** was proposed as 20*E*,23R*-hydroxy-nor-cholest-4,20-dien-3-one.

The HRESIMS data of **3** showed the sodium-adduct ion signal at *m*/*z* 451.3187, implying a molecular formula of C_28_H_44_O_3_ (calcd. [M + Na]^+^ *m*/*z* 451.3188) (Supplementary Materials, Figure S17). The ^1^H and ^13^C NMR spectra of **3** revealed characteristic resonances of a C_28_ cholestane skeleton (Supplementary Materials, Figures S18 and S19). Similar to the NMR data of **2**, the ^1^H NMR spectrum of **3** showed a singlet of an olefinic proton at *δ*_H_ 5.74, attributable to a cyclic enone group (Δ^4^-3-ketone) of the A-ring, which is further supported by a conjugated ketocarbonyl resonance at *δ*_C_ 199.7. Specifically, the 1D NMR data of **3** exhibited the presence of an oxygenated methine group (*δ*_H_ 4.65; *δ*_C_ 74.2) and the observed COSY correlations from H_2_-15 (*δ*_H_ 2.27, 1.34) and H-17 (*δ*_H_ 1.25) to H-16 (*δ*_H_ 4.65) suggested the oxygenated methine group (*δ*_H_ 4.65; *δ*_C_ 74.2) is present in D-ring of **3** (Figure 3B). The ^1^H resonances at *δ*_H_ 4.75 and 4.68 was assigned for the exomethylene protons by HSQC correlations (Supplementary Materials, Figure S21), and the HMBC cross-peaks from the two doublets at *δ*_H_ 1.03 of the terminal methyl groups (Me-26 and Me-27) and H_2_-28 (*δ*_H_ 4.75 and 4.68) to C-24 (*δ*_C_ 156.3) located the exocyclic double bond on C-24. Further HMBC correlation from Me-21 (*δ*_H_ 1.32) to C-17 (*δ*_C_ 60.6) established the linkage of this side chain to C-17 and a methyl substitution at an oxygen-bearing quaternary carbon (C-20, *δ*_C_ 76.5). This overall elucidated the steroidal side chain to be a methylidene group on C-24 (Figure 3B). The relative configurations of the stereogenic carbons of **3** were established by NOESY correlations (Figure 3B and Supplementary Materials, Figure S23). The NOESY correlations of the protons of the cholestane skeleton of **3** were also consistent with those of typical cholestane-based derivatives (Figure 3B). The NOESY correlation from H-14 to H-16 supported the existence of the 16β-hydroxy group. The relative configuration of C-20 was also conducted by the application of computational NMR chemical shift calculations supported by DP4 analysis (Figure 4B). Computational data for **3** were obtained from statistical calculations utilizing the aforementioned protocol and level of theory. Conformers (36 for diastereomer a (20*R*) and 25 for b (20*S*) were subsequently generated (Supplementary Materials, Table S4), and their respective chemical shift values were calculated and averaged for DP4 analysis (Supplementary Materials, Table S5). Comparative DP4 probabilities of the two diastereomers concluded 100.0% for diastereomer a considering both ^1^H and ^13^C NMR chemical shifts (Figure 4B and Supplementary Materials, Figure S24). Thus, the structure of **3** was suggested as 16β,20*R*-dihydroxy-cholest-4,24-dien-3-one.

**Figure 5:**
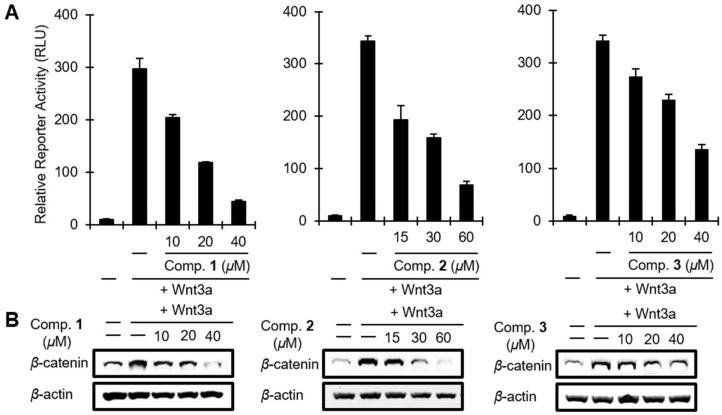
The effect of **1**, **2**, and **3** on the Wnt/β-catenin pathway. (**A**) HEK293-FL reporter cells were incubated with **1**–**3** in the presence of Wnt3a-CM for 15 h and luciferase activity was determined. (**B**) Cytosolic proteins were prepared from HEK293-FL reporter cells treated with the vehicle or **1–3** in the presence of Wnt3a-CM for 15 h and subjected to western blotting.

A large number of studies have reported that abnormal activation of Wnt/β-catenin signaling and overexpression of β-catenin response transcription (CRT) are frequently observed in the development and progression of colorectal cancer [2,3]. To evaluate modulatory activities of **1**, **2**, and **3** on the CRT in this signaling pathway, a genetically engineered HEK293 cell line (HEK293-FL reporter cells) which stably harbored a synthetic β-catenin/Tcf-dependent firefly luciferase (FL) reporter and hFz-1 expression plasmid was used [38]. Chemical effects on the Wnt/β-catenin pathway in these reporter cells have been normalized by the β-catenin/Tcf-driven FL activity with cell viability. As shown in Figure 5A, FL activity was significantly stimulated upon the treatment with Wnt3a-conditioned medium (Wnt3a-CM). Importantly, the supplementation with **1**–**3** at three different micromolar concentrations (10, 20, and 40 μM for **1** and **3**; and 15, 30, and 60 μM for **2**) caused significant inhibition of CRT activity, stimulated by Wnt3a-CM, in a dose-dependent manner (Figure 5A). Under the same condition, these compounds did not affect cell viability in HEK293-FL reporter cells, which have an intact Wnt/β -catenin pathway (Supplementary Materials, Figure S25).

It has been reported that CRT is primarily dependent on the level of intracellular β-catenin which is controlled by the proteasomal degradation route. Normally, β-catenin is phosphorylated at Ser45 and Ser33/37/Thr41 by casein kinase 1 (CK1) and glycogen synthase kinase-3β (GSK-3β), respectively, in a complex with APC and axin (called β-catenin destruction complex), leading to the degradation of β-catenin through a ubiquitin-dependent mechanism [39]. Thus we further examined the effects of **1**–**3** on the protein level of intracellular β-catenin using Western blot analysis. As expected [38], the up-regulation of protein level of β-catenin upon the treatment of Wnt3a-CM (Figure 5B) and clear dose-dependent down-regulation in the cytosolic β-catenin level from the treatment with **1**–**3** were found in the cytosolic proteins prepared from the HEK293-FL reporter cells (Figure 5B). This implies that **1**, **2,** and **3** inhibit Wnt/β-catenin signaling through the down-regulation of cytosolic β-catenin levels.

**Figure 6:**
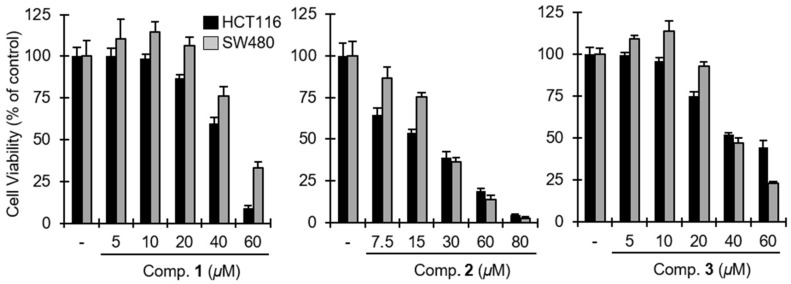
The effect of **1**, **2**, and **3** on the viability of CRT-positive colon cancer cells. the Wnt/β-catenin pathway. HCT116 and SW480 colon cancer cells were incubated with different concentrations of **1**–**3** for 48 h and cell viability was determined by Cell-Titer-Glo assay.

Previous studies have also shown that the suppression of the Wnt/β-catenin pathway inhibits the proliferation of CRT-positive colon cancer cells [39]. Since **1**–**3** reduced cytosolic β-catenin levels, we ultimately evaluated the inhibitory effect of these compounds on the growth of representative CRT-positive colon cancer cells. As a result, **1**, **2**, and **3** exhibited cytotoxic activity in HCT116 colon cancer cells, which contain a Ser45 (CK1 phosphorylation site) deletion mutation in β-catenin, with IC_50_ values of 43.5, 19.7, and 48.0 μM, respectively (Figure 6). In addition, the viability of SW480 colon cancer cells, which display elevated β-catenin expression due to mutation in APC, was decreased by treatment with **1** (IC_50_ = 54.8 μM), **2** (IC_50_ = 24.2 μM), and **3** (IC_50_ = 41.3 μM) (Figure 6). Several lines of evidence in this study indicate that **1**, **2**, and **3** suppress the Wnt/β-catenin pathway through a mechanism independent of β-catenin destruction complex. These compounds were still able to decrease CRT activity and the cytosolic β-catenin level in the presence of Wnt3a, which inactivates β-catenin destruction complex. Furthermore, **1**, **2**, and **3** showed cytotoxic activity in HCT116 and SW480 colon cancer cells, which have alteration in destruction complex-mediated β-catenin degradation. The mechanism underlying downregulation of β-catenin induced CRT of **1**–**3** needs to be further investigated.

## 3. Materials and Methods

### 3.1. General Experimental Procedures

Circular dichroism spectrum was recorded on a Chirascan qCD (Applied Photophysics, Leatherhead, Surrey, UK). Medium-Pressure Liquid Chromatography (MPLC) (Biotage IsoleraTM, Uppsala, Sweden) was performed using a C_18_ SNAP cartridge KP-C18-HS (Biotage, Charlotte, NC, USA) at a flow rate of 30 mL/min. The sample separation was monitored at 205 and 254 nm UV/vis wavelengths. Reversed-phase High-performance liquid chromatography (HPLC) was performed on a Gilson HPLC system (Gilson, Inc. Middleton, WI, USA) with a Phenomenex Luna C_18_ (2) 5 µm column (250 × 21.20 mm) (Phenomenex, Torrance, CA, USA) at a flow rate of 6 mL/min. Thin layer chromatography (TLC) was performed on pre-coated silica gel 60 F254 plates (Merck, Darmstadt, Germany). ^1^H and ^13^C NMR, and 2D (COSY, HSQC, HMBC and NOESY) NMR spectra were recorded on a Bruker AscendTM 600 MHz (Bruker, Billerica, MA, USA). High-resolution Electrospray Ionization mass (HRESIMS) data were obtained utilizing a Synapt G2 Waters mass spectrometer (Waters, Milford, MA, USA).

### 3.2. Sponge Collection

The specimen used for compound extraction and subsequent chemical analyses (2010-AK-49) was collected with a bottom trawl during a fisheries stock assessment survey aboard the RV Sea Storm. The specimen was collected at a depth of 133 m south of Kanaga Island, in the central Aleutian Islands region (51.642° N, 177.453° W), on 25 July 2010. In life, the sponge forms a massive palmate fan with a thick short stem, with deeply incised, ragged margins; the surface is frequently, deeply cracked. The texture of the stem is tough and incompressible. The fan is fibrous and flexible. Colour in life is light orange-yellow. The sponge is an undescribed species of *Monanchora* (Order Poecilosclerida: Family Crambeidae), identified as *Monanchora* n. sp. 1 (yellow fan) [13]. The original voucher specimen (2010-AK-49) is deposited at the Department of Pharmacognosy, College of Pharmacy, Chungnam University, Korea, and another voucher was deposited at the Natural History Museum, London, United Kingdom (NHMUK2011.2.11.3). A subsample of this NHMUK voucher (NIWA 92956) is accessioned within the NIWA Invertebrate Collection (NIC), Wellington, New Zealand. 

### 3.3. Taxonomic Notes

*Monanchora pulchra* was first described by Lambe [28], from beach-thrown specimens collected from Gull and Unalaska Islands, in the Aleutian Islands Archipelago. The species has since been collected from other island ridges and passes in the Aleutian Islands [40]. In a review of trawl-caught specimens from the Aleutian Island region in 2010 a new, undescribed species, *M*. n. sp. 1 (yellow fan) was reported with a listing of key differences between the two species [13]. Subsequent careful study of numerous specimens collected from the region prior to 2010 with submersibles [41] revealed yet a third group referred to as *M*. cf. *pulchra*. Study of the gross morphology and bathymetric distribution of the three groups of sponges enhances our ability to target future collections and determine if the active metabolites are species-specific; the shape and thickness of the sponge lamella and the colour in life, appear to be reasonably specific to each taxon, and there is some bathymetric differentiation evident as well. 

The first taxon is *M. pulchra*, described by Lambe (Supplementary Materials, Figure S26) [28], as a thin, yellowish fan with deeply incised margins and narrow surface aquiferous canals visible in life, large style megascleres (1100 µm), small robust microscleres (unguiferous arcuate chelae) with longish teeth-like alae, and sigma microscleres. The second taxon is referred to as *M*. cf. *pulchra* (Supplementary Materials, Figure S27) because the specimens generally resemble the original species, but form thin, leafy, pumpkin- to dark orange-colored fans, with oscules along the margins and on one side. The spiculation differs in key areas as well: the style megascleres are smaller than those of *M. pulchra* sensu stricto (900 µm long) and the microsclere chelae are smaller, thinner and predominantly anchorate in form; they are only occasionally unguiferous arcuate in form (as in *M. pulchra* sensu stricto) with numerous very short alae. These sponges were collected consistently in relatively shallow water (< 100 m). The third group, identified here as *Monanchora* n. sp. 1 (large isochelae and styles), differs considerably from *M. pulchra* sensu stricto (Supplementary Materials, Figure S28) in that the specimens may be digitate or more commonly form stalked, thick fans with a deeply cracked surface. The surface appears to be more densely siliceous and opaque in life, than in the other groups. The style megascleres are large and very similar to those in *M. pulchra* sensu stricto, but the key difference is the possession of large, robust, anchorate/arcuate chelae with numerous very short alae. This third group of specimens, and the single specimen of *M. pulchra* sensu stricto collected, were all captured in deeper waters around 150 m. One of our future objectives is to determine whether the active compounds are found in all three closely-related taxa, and in what proportions. As the compounds of interest are not abundant, it may be more expedient to pool all specimens of *Monanchora* for extraction rather than embarking on the detailed determination of each individual’s identification.

### 3.4. Ecological Observations

Review of video footage of the seafloor collected throughout the central Aleutian Islands with submersibles in 2002–2004, coupled with voucher specimen collections, indicated that *M. pulchra* was widely distributed, locally abundant, and found at depths between 79 and 330 m [41]. Taxonomic analyses of collected specimens, all from depths < 100 m, indicated that all were *M. pulchra*. Our new analysis of additional collected specimens indicates that there are three distinct taxa or groups and that they may occupy different depths and habitats. *Monanchora* cf. *pulchra* appears to occupy rougher habitats including bedrock outcrops at depths principally shallower than 100 m. The other two taxa, *M. pulchra* and *Monanchora* n. sp. 1, appear to occupy lower-profile habitat consisting of sand, pebbles, and small cobbles and at depths > 100 m. The latter habitat type is accessible to trawl sampling, hence the collection of those specimens by trawl gear. All species occupy habitats that are subjected to moderate to high water currents.

### 3.5. Organic Extraction and Compound Isolation

*Monanchora* sp. (~1 kg) was cut into small pieces and extracted with MeOH to yield a dark colored-solid extract (53.2 g). The MeOH extract (2.5 g) was subjected to C_18_ reversed-phase MPLC (Biotage SNAP Cartridge, KP-C18-HS, 120 g) using isocratic elution with MeCN-H_2_O (40%–100% MeCN, total volume 4 L) at a flow rate of 30 mL/min wherein 15 fractions (Frs.1-15) were collected. Fr. 6 (20.0 mg) was further purified by Gilson HPLC system (Phenomenex Luna C_18_ (2) 5 µm 250 × 21.2 mm column; flow rate, 10 mL/min; mobile phase composition MeCN-H_2_O gradient solvent system: 0–10 min, 40% MeCN; 10–15 min, 50% MeCN; 15–25 min, 50% MeCN; 25–30 min, 60% MeCN; 30–60 min, 60% MeCN; 60–65 min, 70% MeCN; 65–85 min, 70% MeCN; 85–90 min, 80% MeCN; 90–130 min; 80% MeCN, 130–135 min; 90% MeCN; 135–150 min, 100% MeCN). Compound **1** (3.0 mg) was eluted at 86 min. Fraction 7 was also purified to yield **2** (*t*_R_ = 107 min, 2.0 mg) and **3** (*t*_R_ = 118 min, 3.0 mg) under the same conditions described above.

Compound (**1**): colorless gum; [α]${}_{D}^{25}$ − 7.8 (*c* 0.09, CH_3_OH); ^1^H and ^13^C NMR data, see Table 1; HRESIMS [M + Na]^+^ *m*/*z* 479.2407 (calculated for C_27_H_36_O_6_Na, 479.2410).

Compound (**2**): colorless gum; [α]${}_{D}^{25}$ + 91.3 (*c* 0.08, CH_3_OH); ^1^H and ^13^C NMR data, see Table 2; HRESIMS [M + Na]^+^ *m*/*z* 407.2926 (calculated for C_26_H_40_O_2_Na, 407.2926).

Compound (**3**): colorless gum; [α]${}_{D}^{25}$ + 62.4 (*c* 0.1, CH_3_OH); ^1^H and ^13^C NMR data, see Table 2; HRESIMS [M + Na]^+^ *m*/*z* 451.3187 (calculated for C_28_H_44_O_3_Na, 451.3188).

### 3.6. Computational Details for ECD Simulation

A truncated structure of **1** (1a, Supplementary Materials, Figure S8) was proposed to minimize computational complexity and expense, and conformational searches with 1a were performed using MacroModel with the MMFF force field (gas phase), a 10 kcal/mol upper energy limit and 0.001 kJ (mol Å)^−1^ convergence threshold on the rms gradient. Redundant conformers were eliminated utilizing a 0.5 Å root-mean-square deviation (RMSD) cut-off for the atoms indicated in red (Supplementary Materials, Figure S8), ultimately leading to identify eight unique conformers. The geometries of these conformers were optimized at B3LYP/6-31G(d) basis set in the polarizable continuum solvation model (PCM) with a dielectric constant representing MeOH. The optimized conformers were then proceeded to ECD calculations at B3LYP/6–31G(d,p) (PCM, MeOH), and the generated excitation energies and rotational strengths were Boltzmann-averaged on the basis of calculated Gibbs free energy (Supplementary Materials, Table S1) and visualized utilizing SpecDis.

### 3.7. Computational NMR Chemical Shifts Calculations for DP4 Analysis

Conformational searches were performed using the MacroModel (Version 9.9, Schrödinger LLC, New York, NY, USA) program interfaced in Maestro (Version 9.9, Schrödinger LLC) with a mixed torsional/low-mode sampling method. Advanced conformational searches were carried out in the MMFF force field, in the gas phase with a 50 kJ/mol energy window and 10,000 maximum iterations based on the original authors’ recommendations [29]. NMR chemical shift calculations of all conformers within 10 kJ/mol of the relative energy were implemented at the Gaussian 09 package (Gaussian Inc., Wallingford, CT, USA) without geometry optimization in the B3LYP/6-31G(d,p) theory level. Chemical shifts values were calculated via an equation below where $\delta_{calc}^{x}$ is the calculated NMR chemical shift for nucleus *x*, $\sigma^{o}$ is the shielding tensor for the proton and carbon nuclei in tetramethylsilane calculated at the B3LYP/6-31G(d,p) basis set. For the consistency of computational outcomes, the geometry of tetramethylsilane was optimized at the aforementioned theory level.

$$\delta_{calc}^{x}=\frac{\sigma^{o}-\sigma^{x}}{1-\sigma^{o}/10^{6}}$$

The DP4 probability analysis was conducted using an applet available at http://www-jmg.ch.cam.ac.uk/tools/nmr/DP4/.

### 3.8. Cell Culture and Reporter Assays

HEK293 (human embryonic kidney cell), HCT116 (human colon carcinoma cell), SW480 (human colon adenocarcinoma cell), and Wnt3a-secreting L cells were obtained from the American Type Culture Collection (Manassas, VA, USA). Each cell lines were cultivated in Dulbecco’s modified Eagle’s medium (DMEM) supplemented with 10% fetal bovine serum (FBS) and two antibiotics (120 µg/mL penicillin, and 200 µg/mL streptomycin). HEK293-FL reporter (TOPFlash) and control (FOPFlash) cells were constructed and Wnt3a-conditioned medium (Wnt3a-CM) was prepared as reported previously [38]. Luciferase assay was conducted using the Dual Luciferase Assay Kit (Promega, Madison, WI, USA) according to the manufacturer’s instructions.

### 3.9. Western Blotting

The cytosolic fraction was prepared as previously described [42]. Proteins were separated by SDS-polyacrylamide gel electrophoresis (PAGE) in a 4 to 12% gradient gel (Invitrogen, Carlsbad, CA, USA) and transferred to nitrocellulose membranes (Bio-Rad Laboratories, Hercules, CA, USA). The membranes were blocked with 5% nonfat milk and probed with anti-β-catenin (BD Transduction Laboratories, Lexington, KY, USA) and anti-actin antibodies (Cell Signaling Technology, Danvers, MA, USA). The membranes were then incubated with horseradish-peroxidase-conjugated anti-mouse IgG (Santa Cruz Biotechnology, Dallas, TX, USA) and visualized using the ECL system (Santa Cruz Biotechnology, Dallas, TX, USA).

### 3.10. Cell Viability Assay

Cells were dispensed into 96-well plates followed by the treatment with compounds **1**–**3** and further incubated for 48 h. The cell viability from compounds treatment was measured using CellTiter-Glo assay kit (Promega, Madison, WI, USA) according to the manufacturer’s instructions. All experiments were carried out in triplicate.

## 4. Conclusions

In summary, we have isolated a new spirocyclic ring-containing sesterterpenoid (**1**) and two new cholestane-type steroids (**2** and **3**) from a deep-water Alaska sponge. The sponge was taxonomically characterized to be an undescribed species of *Monanchora*. The chemical structures of these metabolites were elucidated by the interpretation of NMR and MS spectral data, and their absolute configurations were determined by comparison of the experimental and calculated ECD spectra, and computational chemical shifts calculation followed by DP4 analysis. We also demonstrated that the isolated compounds exhibit an antiproliferative effect on two β-catenin response transcription (CRT)-positive colon cancer cell lines, HCT116 and SW480. We further showed that these metabolites inhibit CRT activity by stimulating the degradation of β-catenin. To the best of our knowledge, this is the first report that sesterterpenoid shows inhibitory effects on the Wnt/β-catenin pathway. Among steroidal metabolites, corticosteroids such as glucocorticoid are known to inhibit the Wnt/β-catenin pathway [43]. Collectively, our studies provide further evidence that marine sponges are an untapped natural resource for the discovery of new bioactive metabolites that expand our view on the structural diversity for the development of the anti-cancer lead structures. 

## Figures and Tables

**Table 1:** ^1^H and ^13^C NMR data of **1** (CDCl_3_, 600 and 150 MHz).

No.	*δ*_C_, type		*δ*_H_ (*J* in Hz)	
1	63.0	CH	4.47	dd, 4.7, 3.0
2	139.4	CH	6.62	dd, 5.9, 1.6
3	139.5	C		
4	198.8	C		
5	38.0	CH_2_	2.59	dd, 13.7, 2.6
			2.49	m
6	33.3	CH	2.54	m
7	142.5	C		
8	125.1	CH	5.66	s
9	96.8	C		
10	44.5	CH_2_	2.32	dd, 13.4, 1.5
			1.87	m ^a^
11	80.1	C		
12	42.5	CH_2_	2.15	brd, 13.2
			1.76	m ^a^
13	65.1	CH	4.65	m
14	125.2	CH	5.25	d, 7.6
15	139.0	C		
16	42.9	CH_2_	2.80	m
17	127.5	CH	5.61	m
18	135.2	CH	6.15	d, 15.6
19	142.1	C		
20	115.3	CH_2_	4.90	d, 5.2
21	16.1	CH_3_	1.86	s
22	63.8	CH_2_	4.12	m
23	24.6	CH_3_	1.78	s
24	17.0	CH_3_	1.74	s
25	18.9	CH_3_	1.84	s
26	170.3	C		
27	22.6	CH_3_	1.96	s

^a^ overlapping signal.

**Table 2:** ^1^H and ^13^C NMR data of **2** and **3** (CDCl_3_, 600 and 150 MHz).

No.	2				3			
	*δ*_C_, type		*δ*_H_ (*J* in Hz)		*δ*_C,_ type		*δ*_H_ (*J* in Hz)	
1	35.8	CH_2_	2.02	ddd, 13.4, 5.1, 3.2	35.8	CH_2_	2.01	m
			1.67	m ^a^			1.68	m
2	33.1	CH_2_	2.40	dd, 14.6, 5.1	34.1	CH_2_	2.41	m
			2.28	ddd, 14.6, 4.3, 2.5			2.34	m
3	199.8	C			199.7	C		
4	124.0	CH	5.73	s	124.1	CH	5.74	s
5	171.6	C			171.1	C		
6	34.1	CH_2_	2.43	m ^a^	32.9	CH_2_	2.40	m
			2.36	dt, 17.0, 3.8			2.27	m
7	32.1	CH_2_	1.84	m ^a^	32.0	CH_2_	1.83	m
			1.03	dd, 12.7, 3.3			1.00	m
8	35.9	CH	1.52	m ^a^	34.7	CH	1.65	m
9	54.2	CH	0.94	m ^a^	54.0	CH	0.93	m
10	38.8	C			38.7	C		
11	21.1	CH_2_	1.58	m ^a^	20.9	CH_2_	1.52	m
			1.42	dd, 13.0, 4.0				
12	38.5	CH_2_	1.84	m ^a^	40.4	CH_2_	2.17	m
			1.17	m ^a^			1.17	m
13	44.0	C			43.1	C		
14	55.5	CH	1.08	m ^a^	53.9	CH	0.87	m
15	24.4	CH_2_	1.73	m ^a^	37.5	CH_2_	2.27	m
			1.23	m ^a^			1.34	m
16	24.8	CH_2_	1.84	m ^a^	74.2	CH	4.65	m
17	59.1	CH	2.08	t, 9.5	60.6	CH	1.25	m
18	13.4	CH_3_	0.63	s	15.1	CH_3_	1.21	s
19	17.6	CH_3_	1.18	s	17.5	CH_3_	1.21	s
20	138.5	C			76.5	C		
21	17.8	CH_3_	1.70	s	26.7	CH_3_	1.32	s
22	128.1	CH	5.24	d, 8.8	42.6	CH_2_	1.68	m
23	73.7	CH	4.15	dd, 8.8, 6.7	28.7	CH_2_	2.03	m
24	34.7	CH	1.70	m ^a^	156.3	C		
25	18.5	CH_3_	0.96	d, 6.7	33.9	CH	2.23	m
26	18.3	CH_3_	0.88	d, 6.7	22.1	CH_3_	1.03	d, 7.0
27					22.1	CH_3_	1.03	d, 7.0
28					106.5	CH_2_	4.75	brs
							4.68	s

^a^ overlapping resonances.

## Supplementary Materials

The following are available online at http://www.mdpi.com/1660-3397/16/9/297/s1, Figure S1–S25: HRESIMS, 1D (^1^H and ^13^C) and 2D NMR spectra, computational studies of compounds **1**–**3**, Figure S26–S28: sponge specimen in this study, Table S1: Gibbs Free Energy and Boltzmann Population of **1a** for ECD computation, Table S2: The major conformers of diastereomers of compound **2**, Table S3: Experimental and calculated NMR chemical shift values (ppm) of compound **2** with diastereomers (diastereomers a and b are described in the main text), Table S4: The major conformers of diastereomers of compound **3**, Table S5: Experimental and calculated NMR chemical shift values (ppm) of compound **3** with diastereomers (diastereomers a and b are described in the main text).
