# Supplementary material for: Sesterterpenoid and Steroid Metabolites from a Deep-Water Alaska Sponge Inhibit Wnt/β-Catenin Signaling in Colon Cancer Cells
Source: Mar Drugs. 2018 Aug 27;16(9):297. doi: 10.3390/md16090297 (PMC6164309; doi:10.3390/md16090297)
Supplement: Supplementary file 1 [file marinedrugs-16-00297-s001.pdf]

Supplementary materials for

Sesterterpenoid and Steroid Metabolites from a Deep-Water Alaska Sponge Inhibit Wnt/β-Catenin Signaling in Colon Cancer Cells

Hyun Bong Park [1,2,†], Nguyen Quoc Tuan [3,4,†], Joonseok Oh [1,2], Younglim Son [5], Mark T. Hamann [6], Robert Stone [7], Michelle Kelly [8], Sangtaek Oh [5,*] and MinKyun Na [3,*]

[1]Department of Chemistry, Yale University, New Haven CT 06520, USA

[2]Chemical Biology Institute, Yale University, West Haven CT 06516, USA

[3]Department of Pharmacognosy, College of Pharmacy, Chungnam National University, Daejeon 34134, Korea

[4]Phúthọ College of Pharmacy, Viettri City, Phúthọ Province 293500, Vietnam

[5]Department of Bio and Fermentation Convergence Technology, BK21 PLUS program, Kookmin University, Seoul 136-702, Korea

[6]Department of Drug Discovery and Biomedical Sciences, Medical University of South Carolina, Charleston SC 29425, USA

[7]Auke Bay Laboratories, Alaska Fisheries Science Center, NOAA National Marine Fisheries Service, Juneau, AK 99801, USA

[8]Coast and Oceans National Centre, National Institute of Water and Atmospheric Research, Auckland Central 1149, New Zealand

*Correspondence: ohsa@kookmin.ac.kr; mkna@cnu.ac.kr

[†]These authors contributed equal to this work.

Index

Supplementary Figures S1 - S28 .... Pages S2 - S25

Supplementary Tables S1 – S5 .... Pages S26 - S34

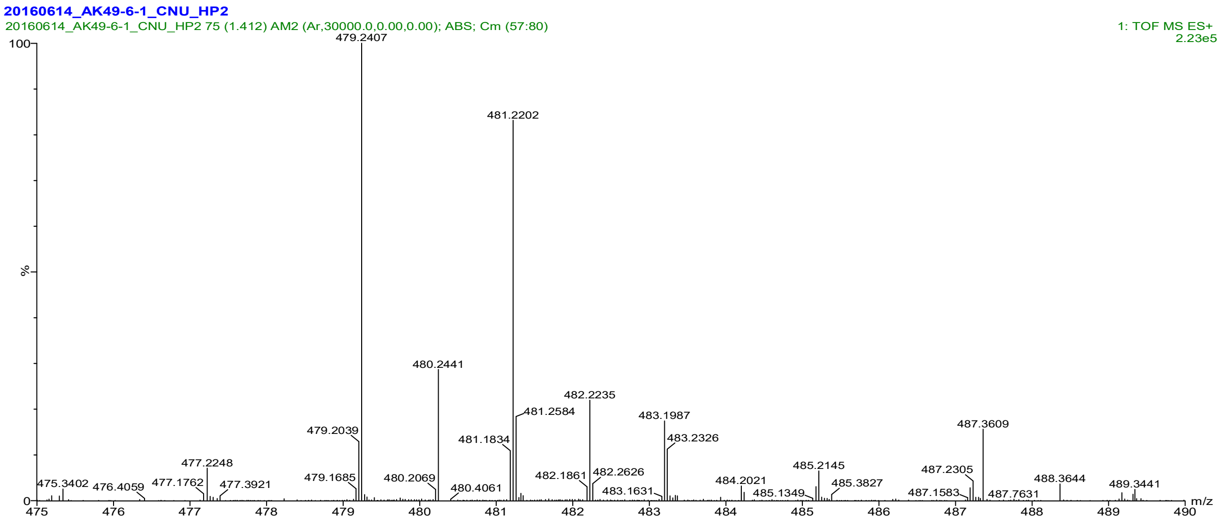

Figure S1. HR-ESI-QTOF-MS spectrum of compound **1**

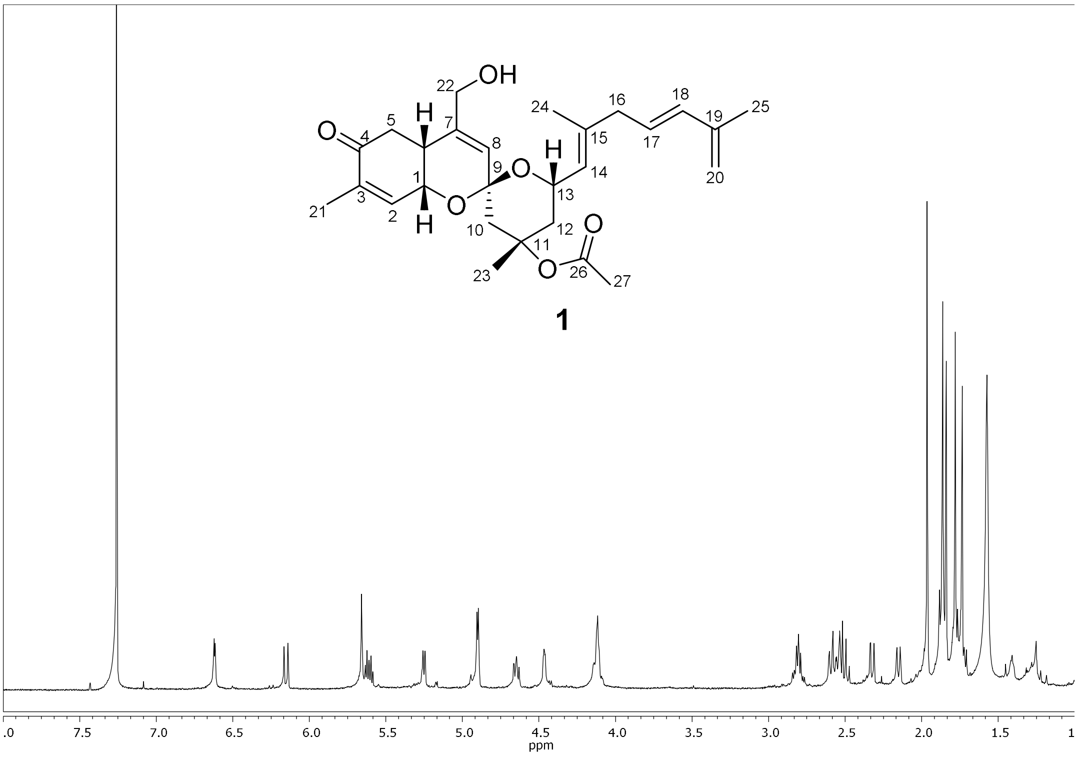

Figure S2. ^{1}H NMR spectrum of compound **1** in $CDCl_3$

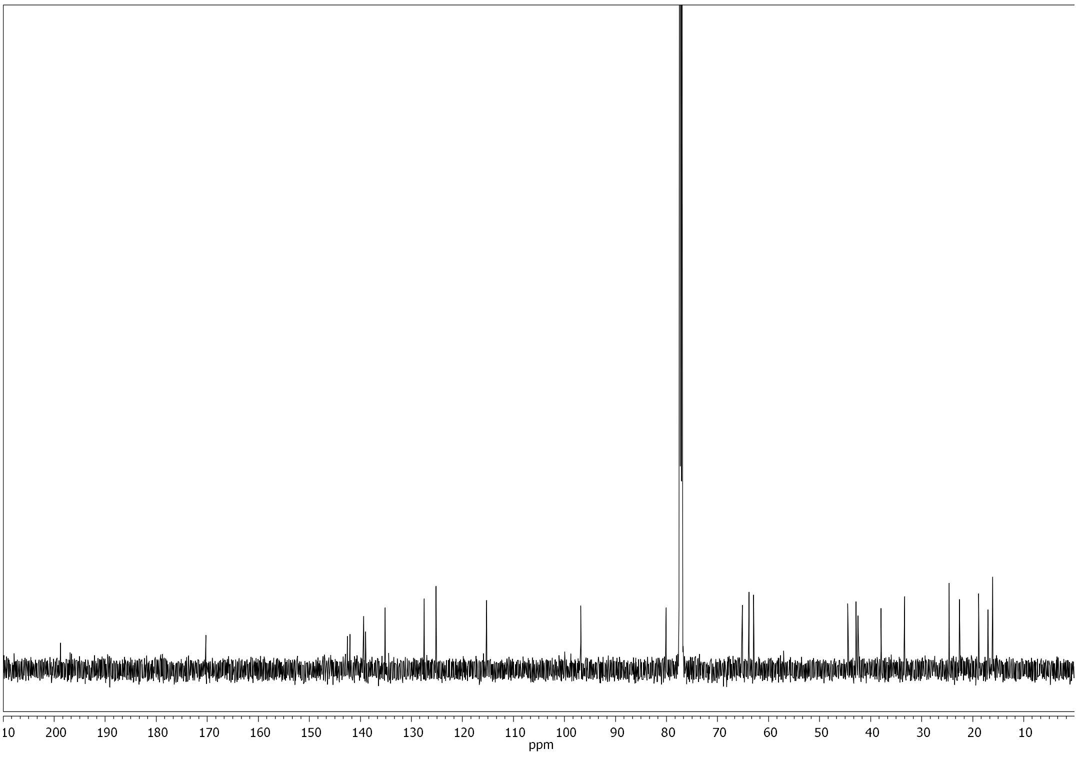

Figure S3. ^{13}C NMR spectrum of compound **1** in $CDCl_3$

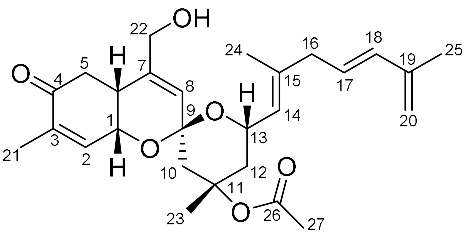

1

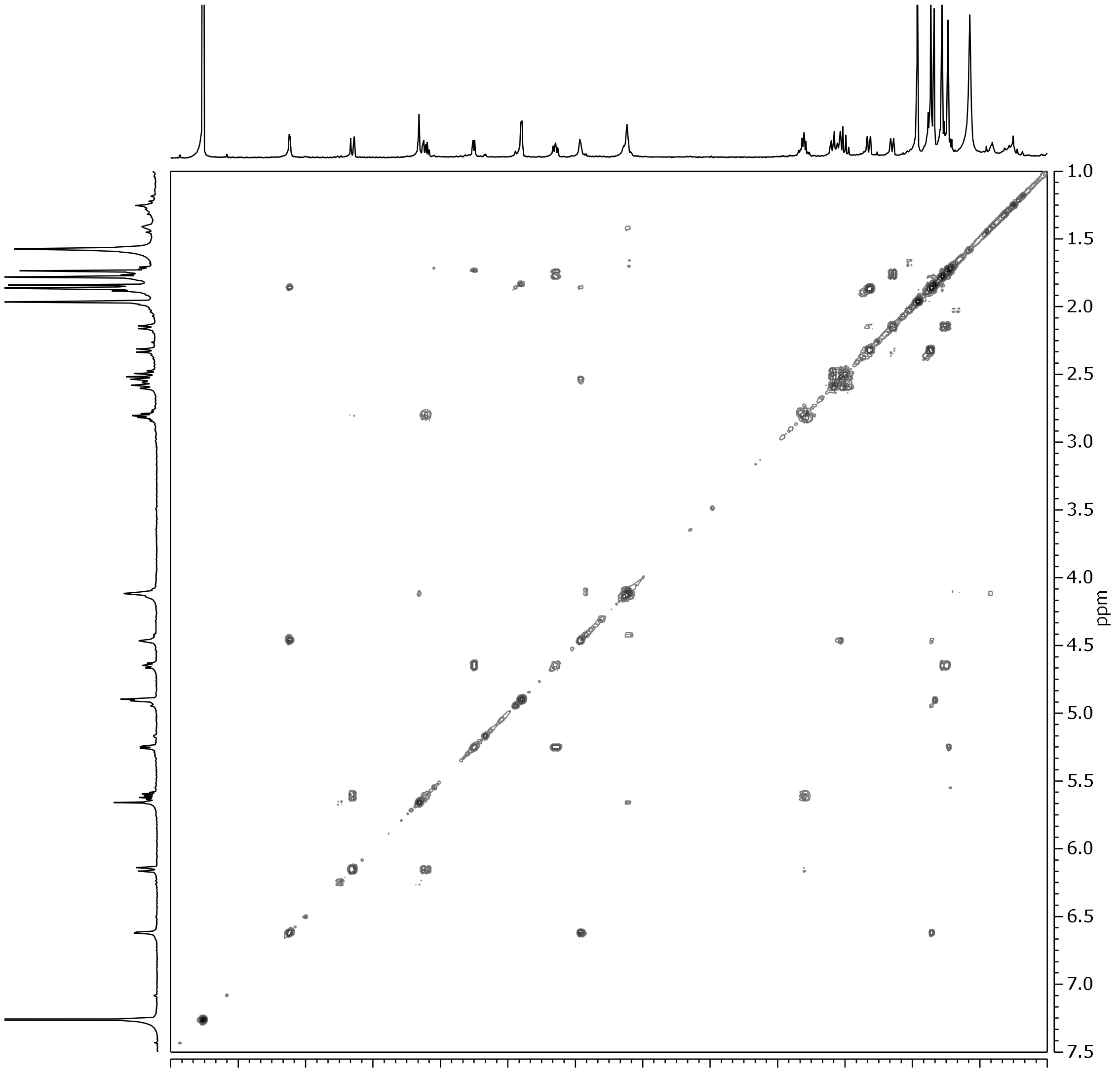

Figure S4. COSY NMR spectrum of compound **1** in $CDCl_3$

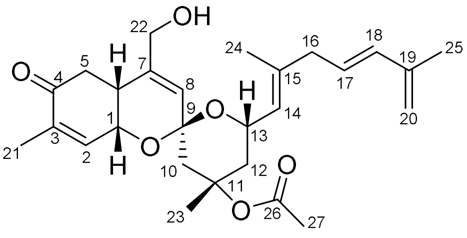

1

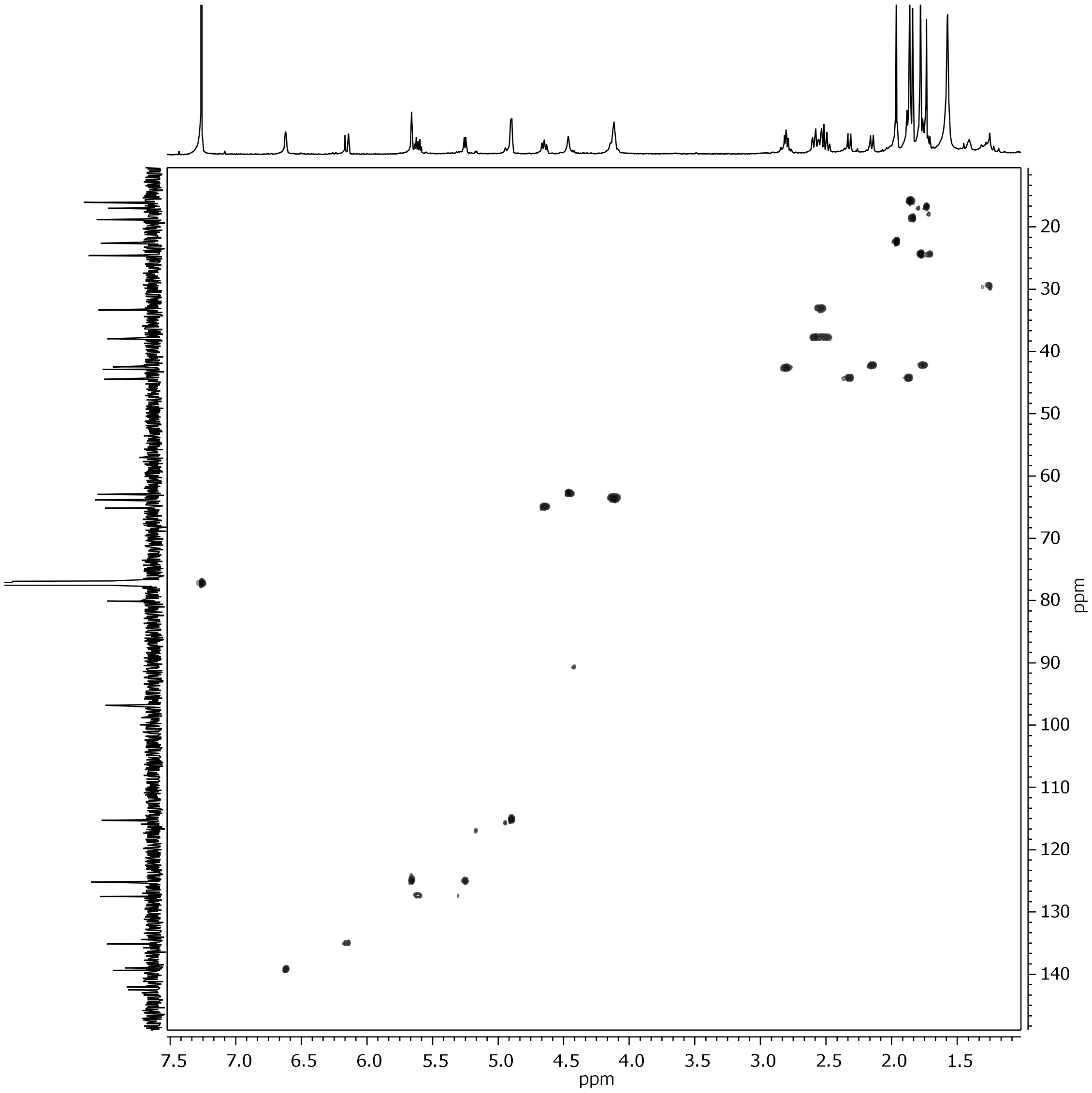

Figure S5. HSQC NMR spectrum of compound **1** in $CDCl_3$

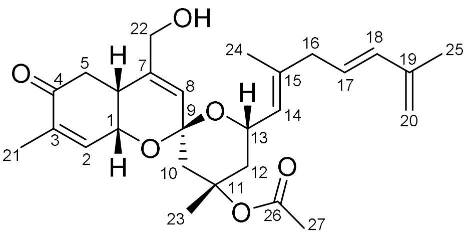

1

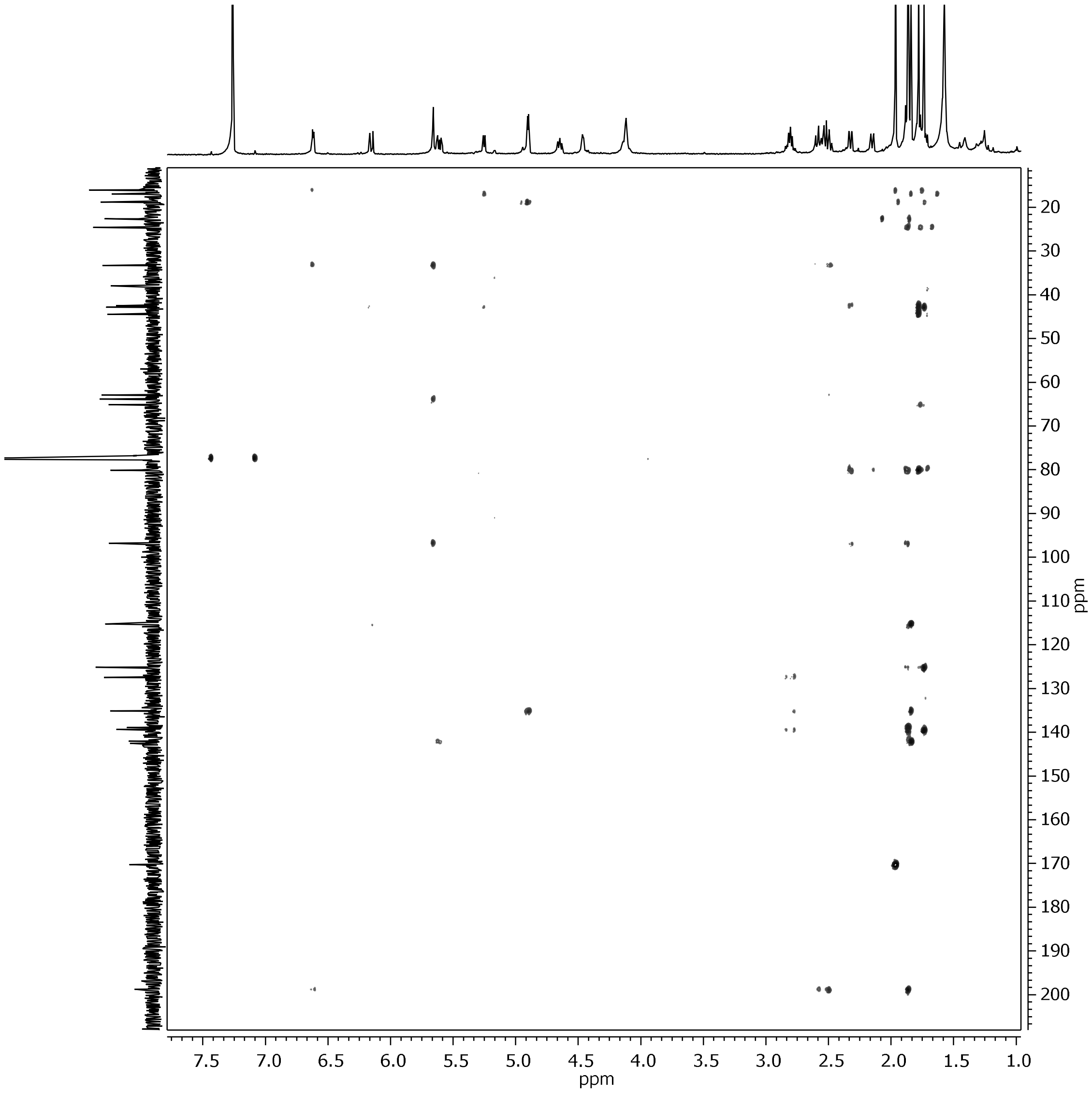

Figure S6. HMBC NMR spectrum of compound **1** in $CDCl_3$

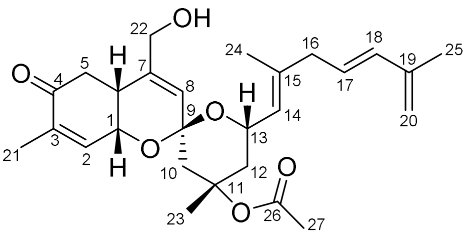

1

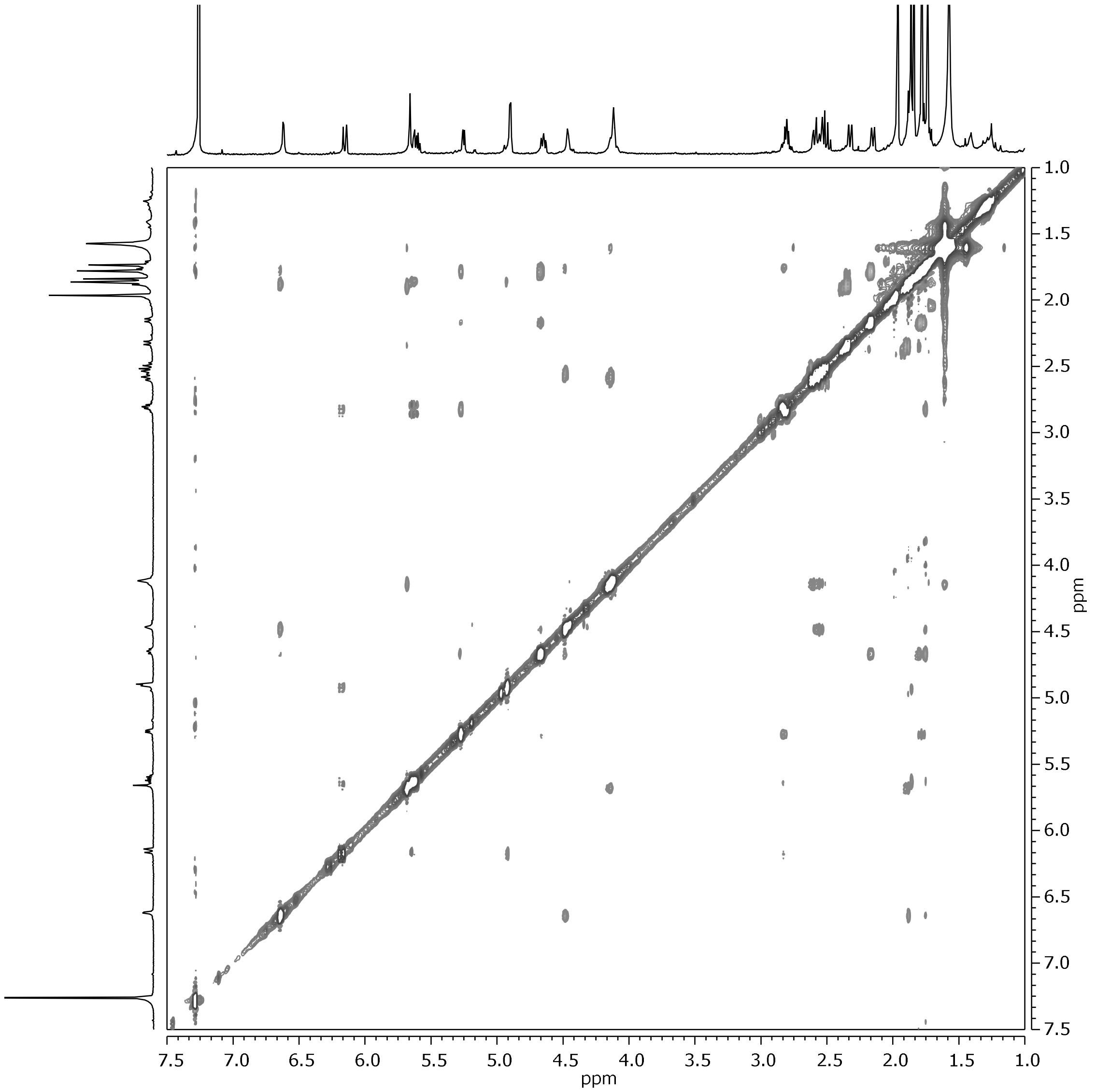

Figure S7. NOESY NMR spectrum of compound **1** in $CDCl_3$

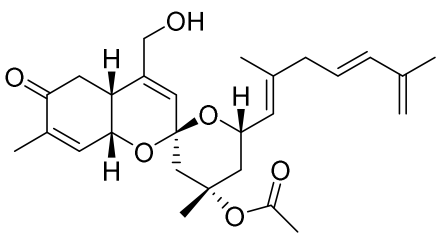

1

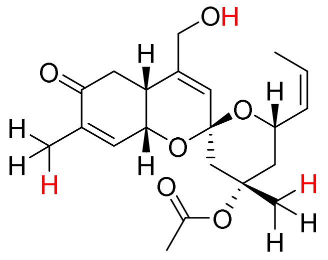

1a

Figure S8. Compound **1** and its truncated structure **1a** for computational analysis

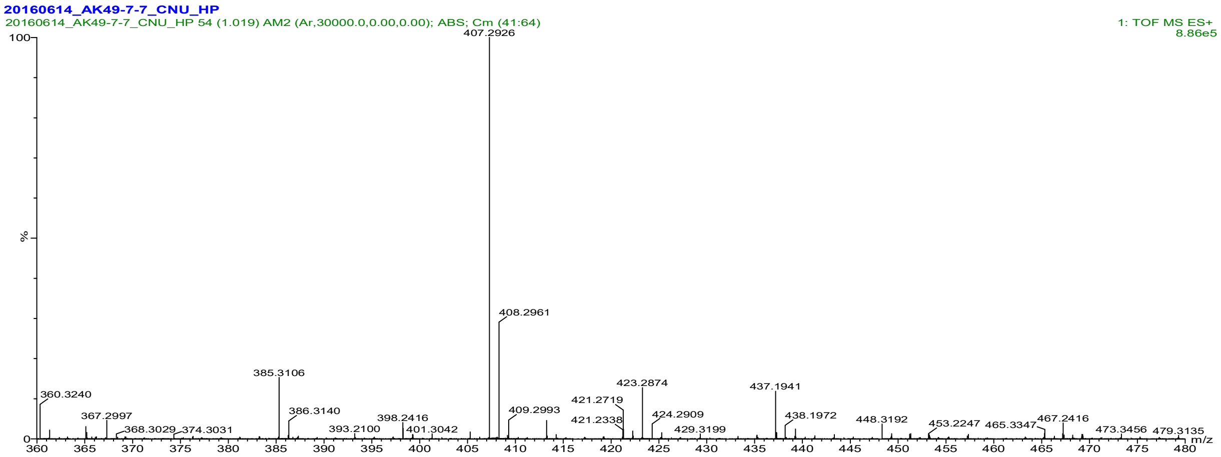

Figure S9. HR-ESI-QTOF-MS spectrum of compound **2**

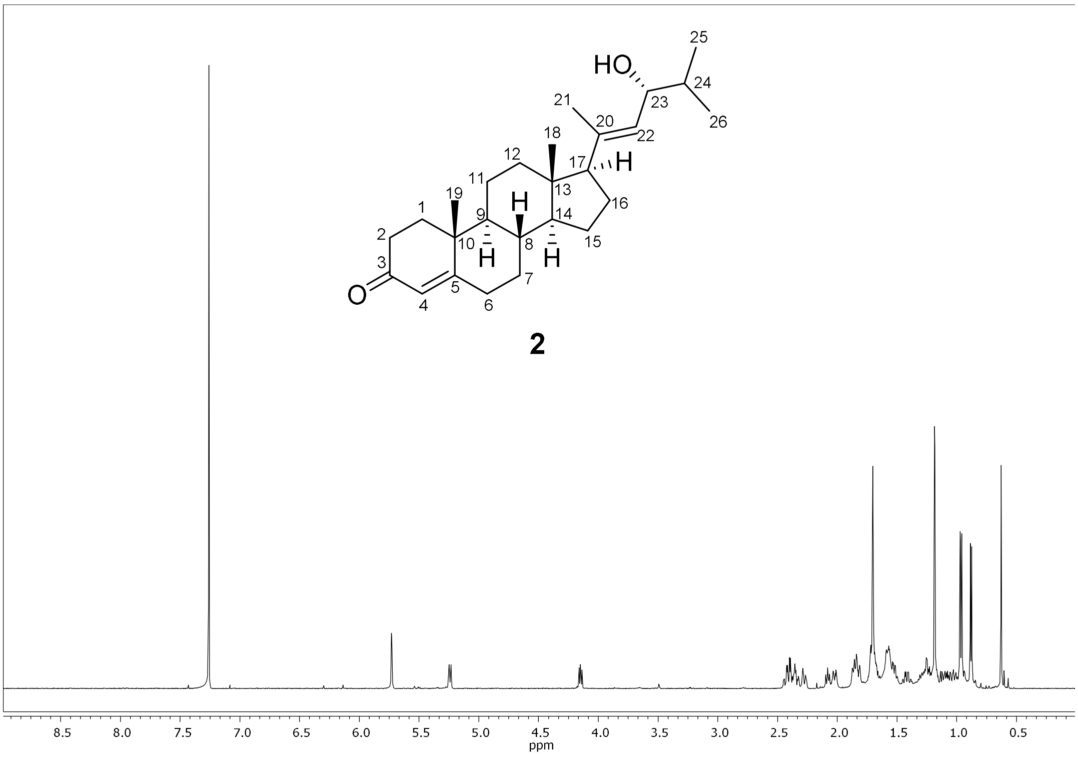

Figure S10. ^{1}H NMR spectrum of compound **2** in $CDCl_3$

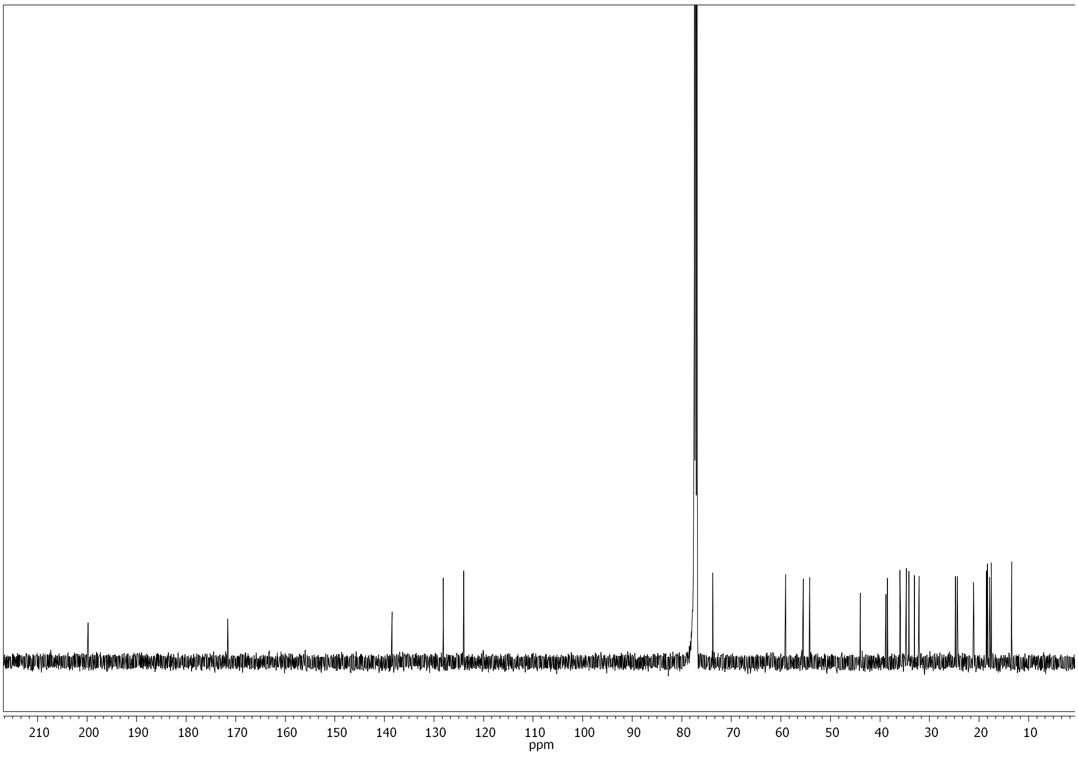

Figure S11. ^{13}C NMR spectrum of compound **2** in $CDCl_3$

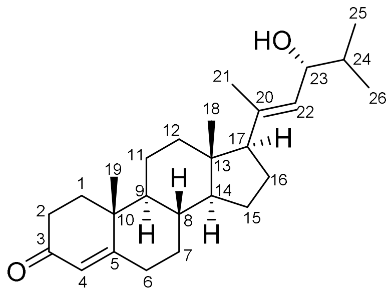

2

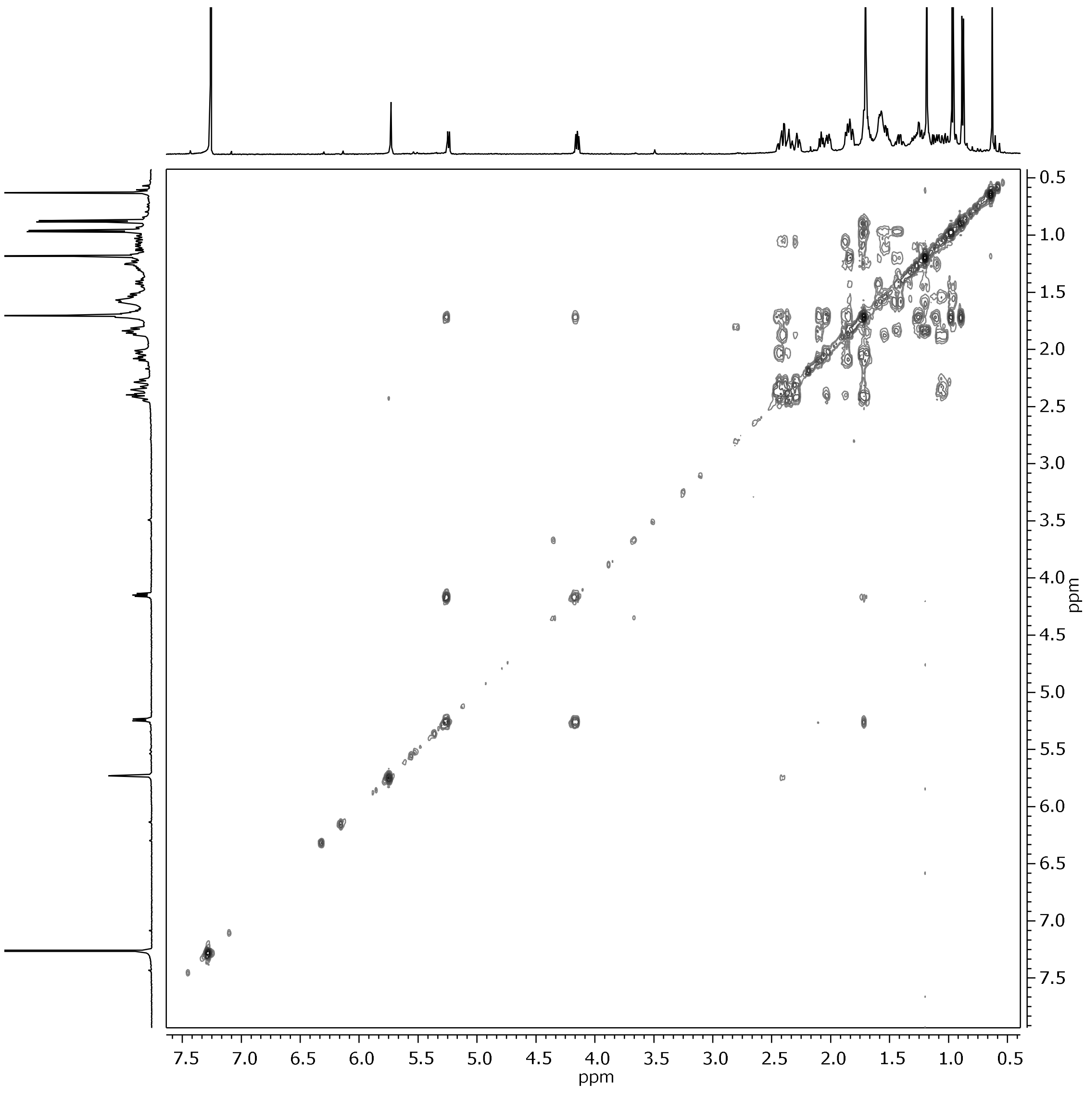

Figure S12. COSY NMR spectrum of compound **2** in $CDCl_3$

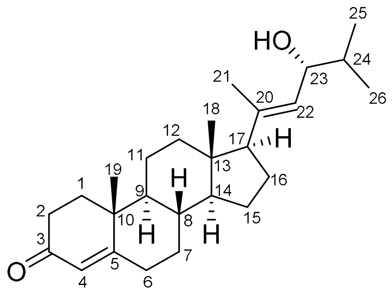

2

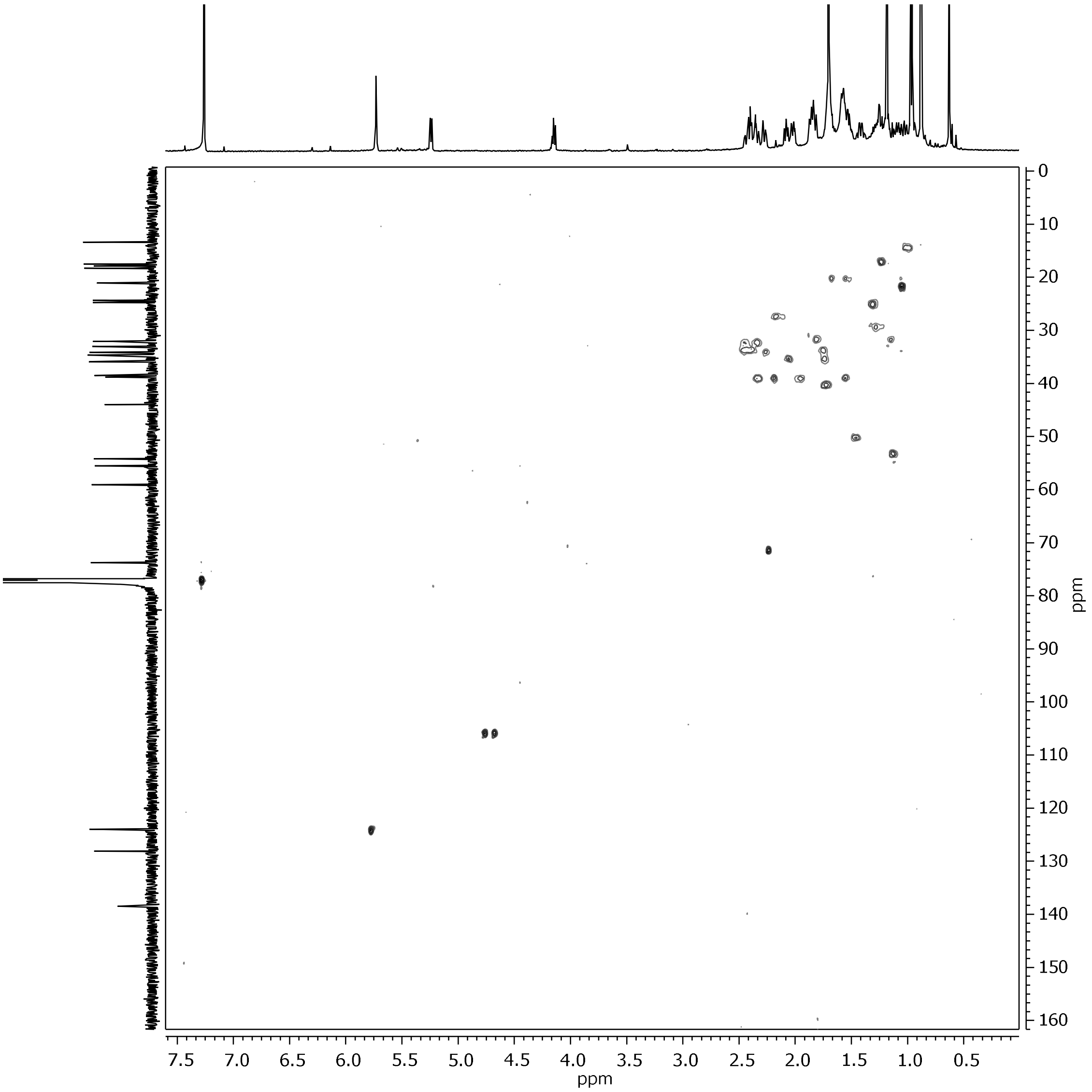

Figure S13. HSQC NMR spectrum of compound **2** in $CDCl_3$

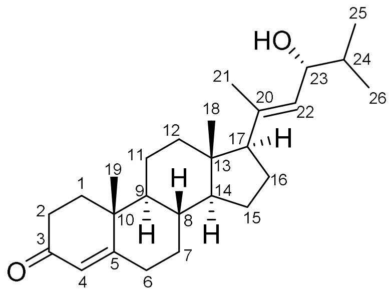

2

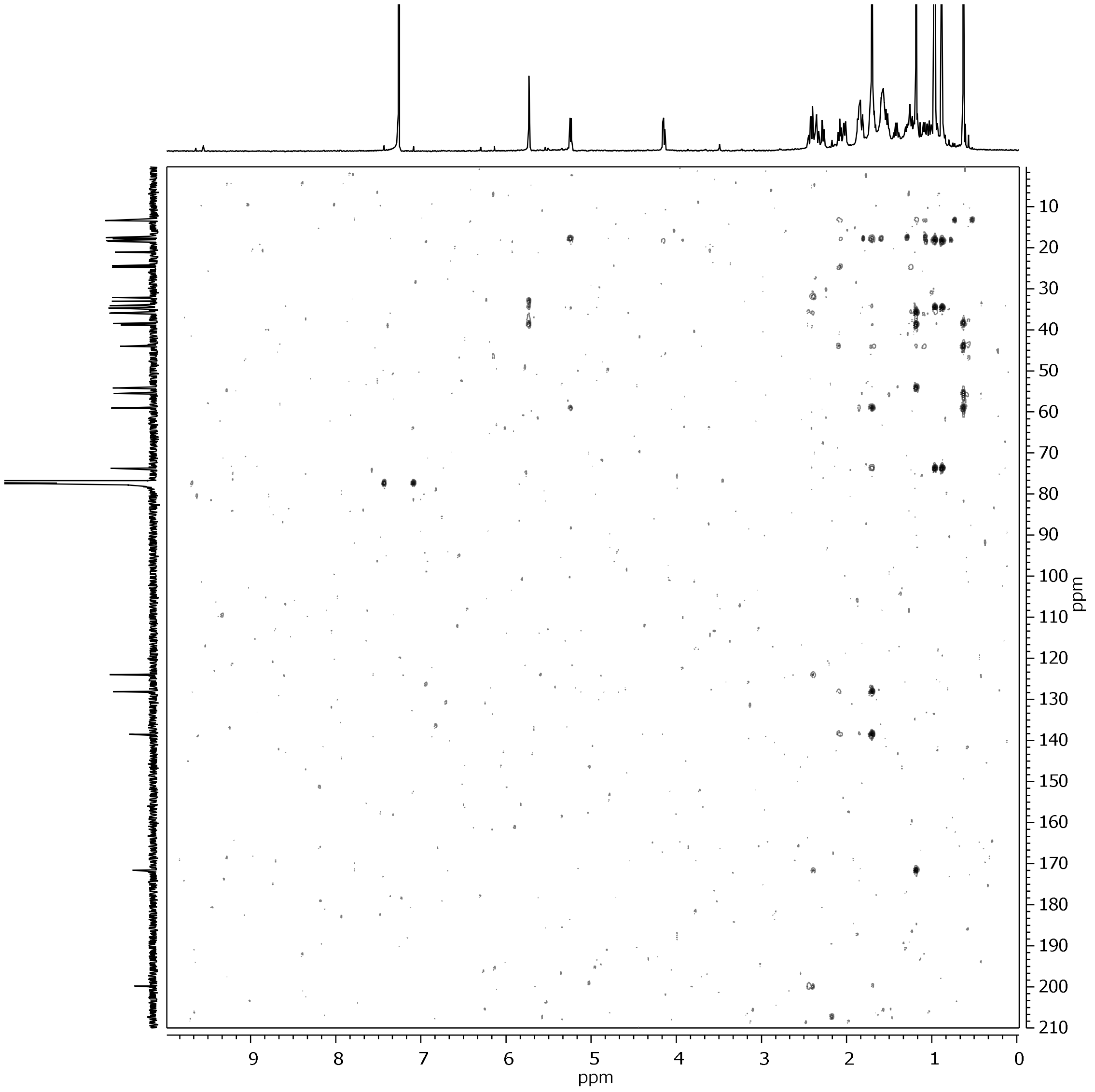

Figure S14. HMBC NMR spectrum of compound **2** in $CDCl_3$

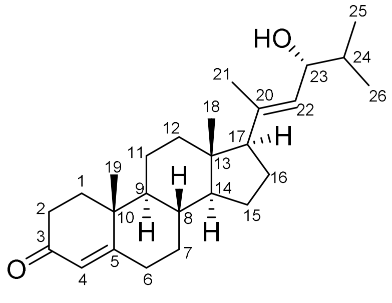

2

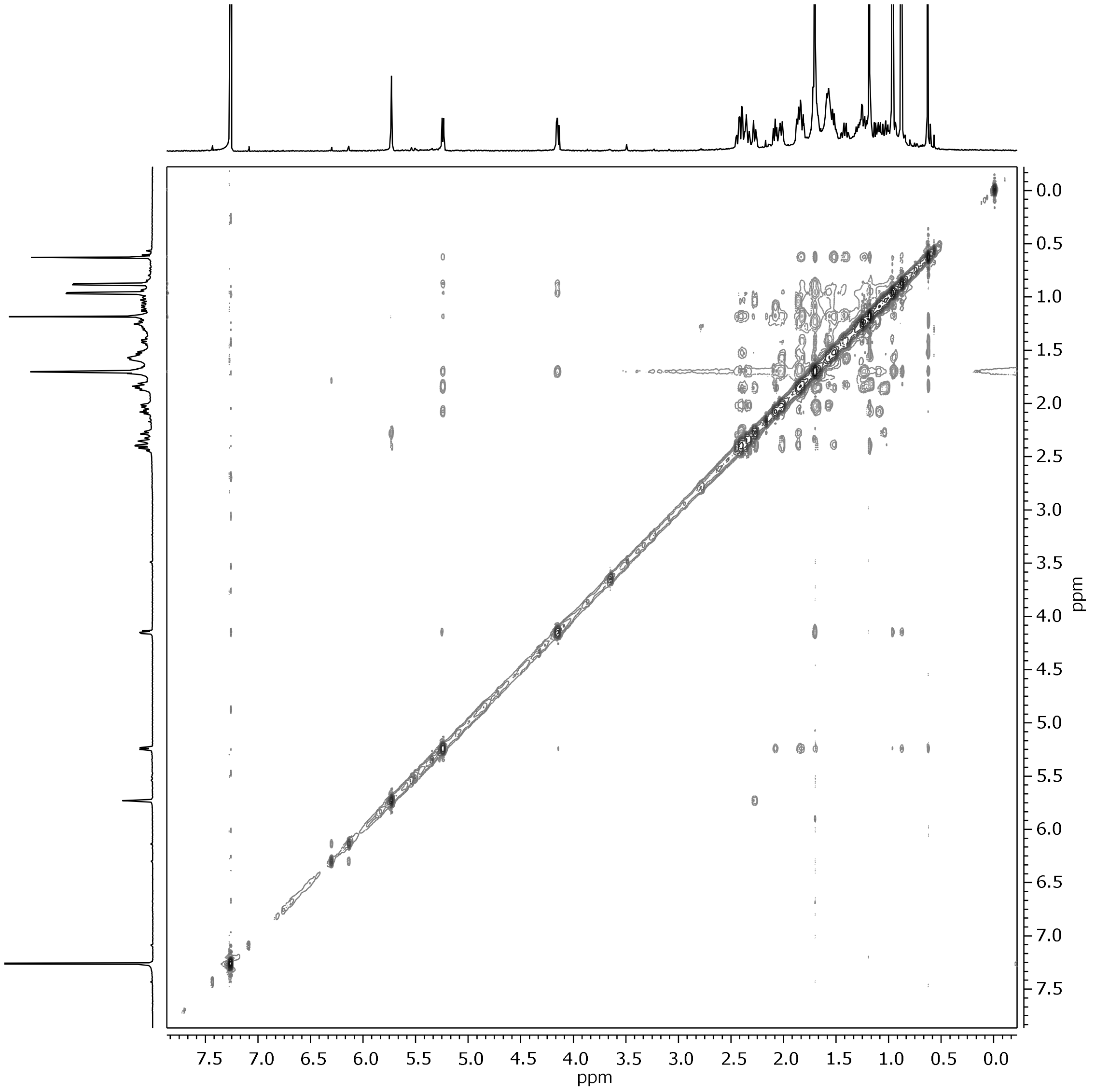

Figure S15. NOESY NMR spectrum of compound **2** in $CDCl_3$

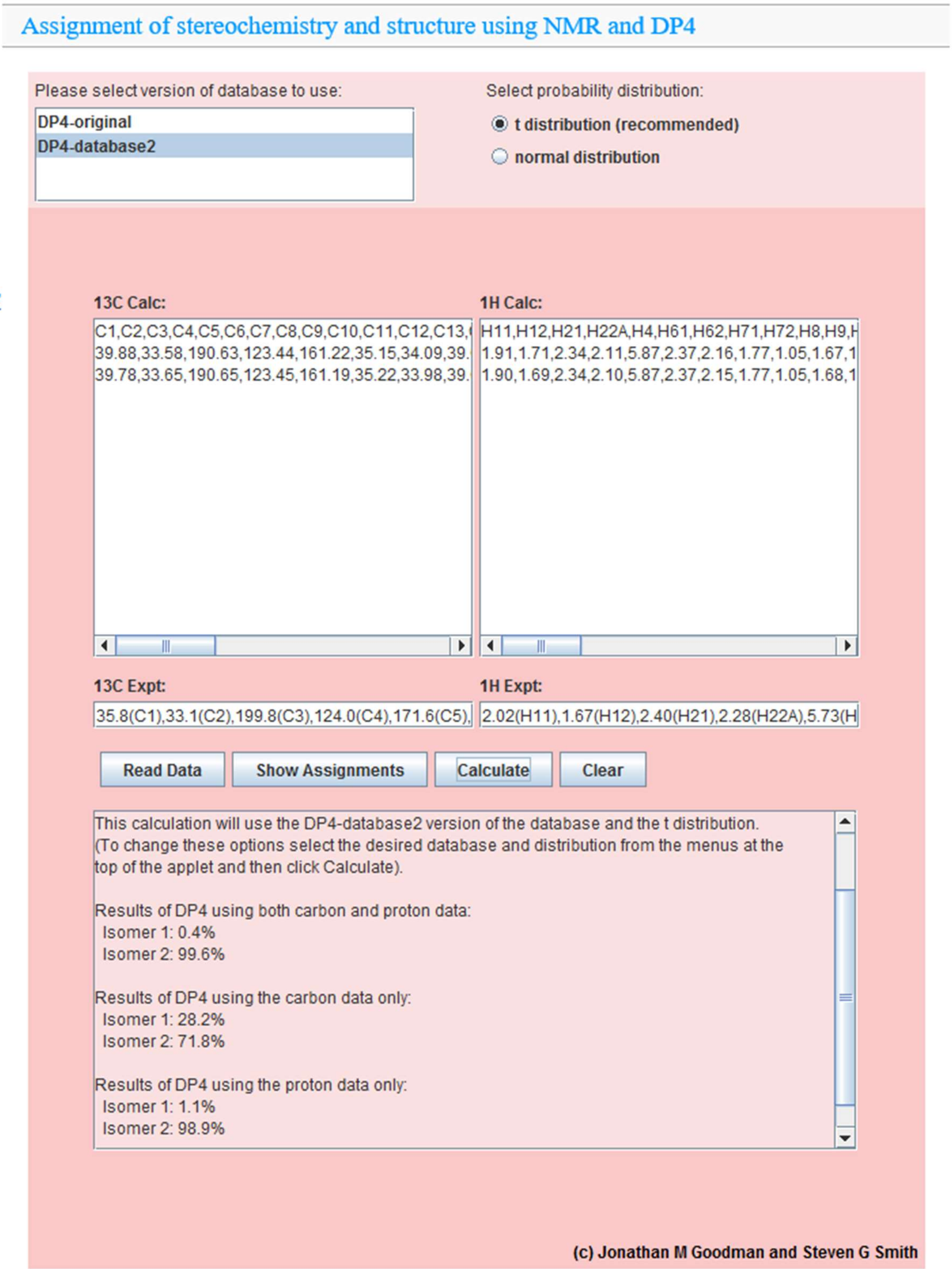

Figure S16. DP4 analysis of compound **2**

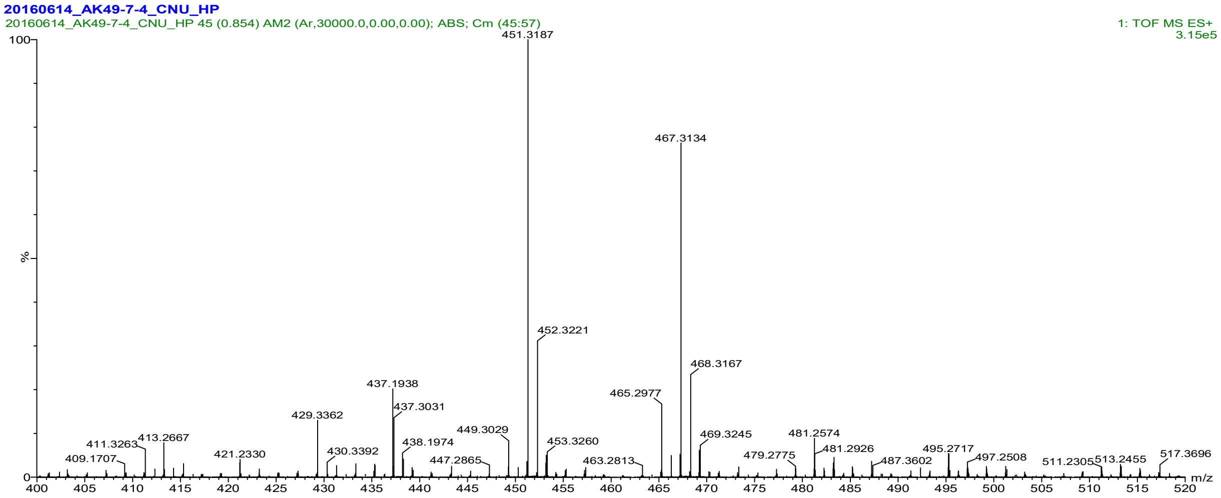

Figure S17. HR-ESI-QTOF-MS spectrum of compound **3**

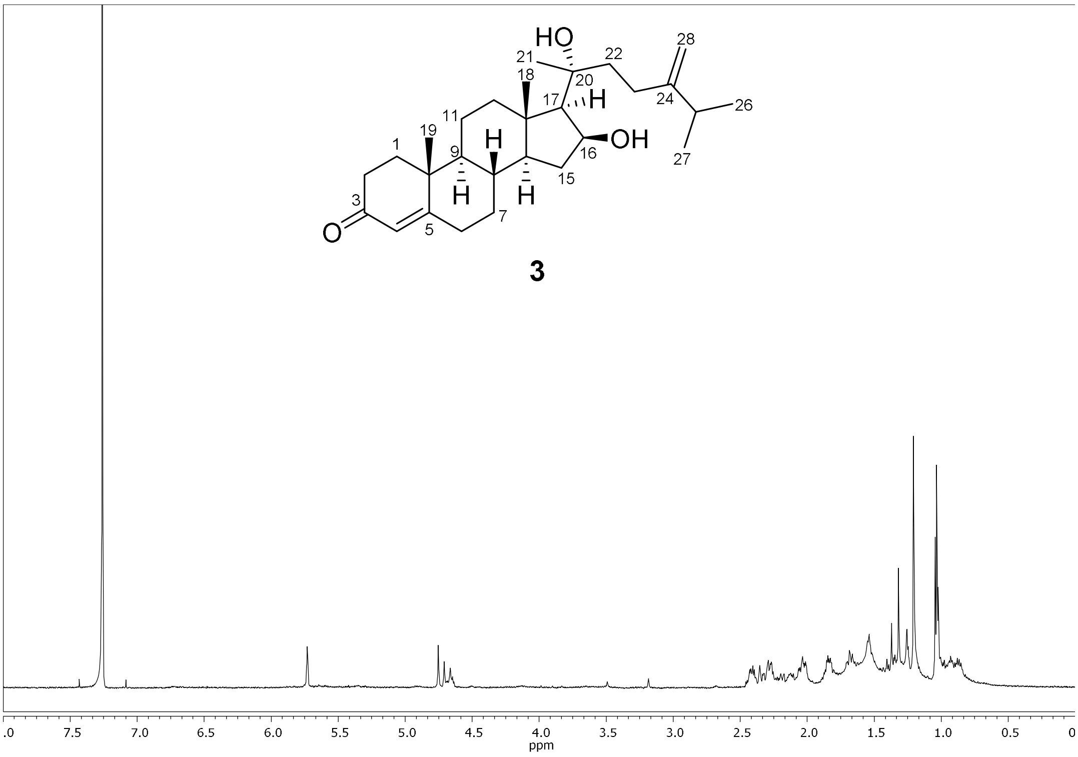

Figure S18. ^{1}H NMR spectrum of compound **3** in $CDCl_3$

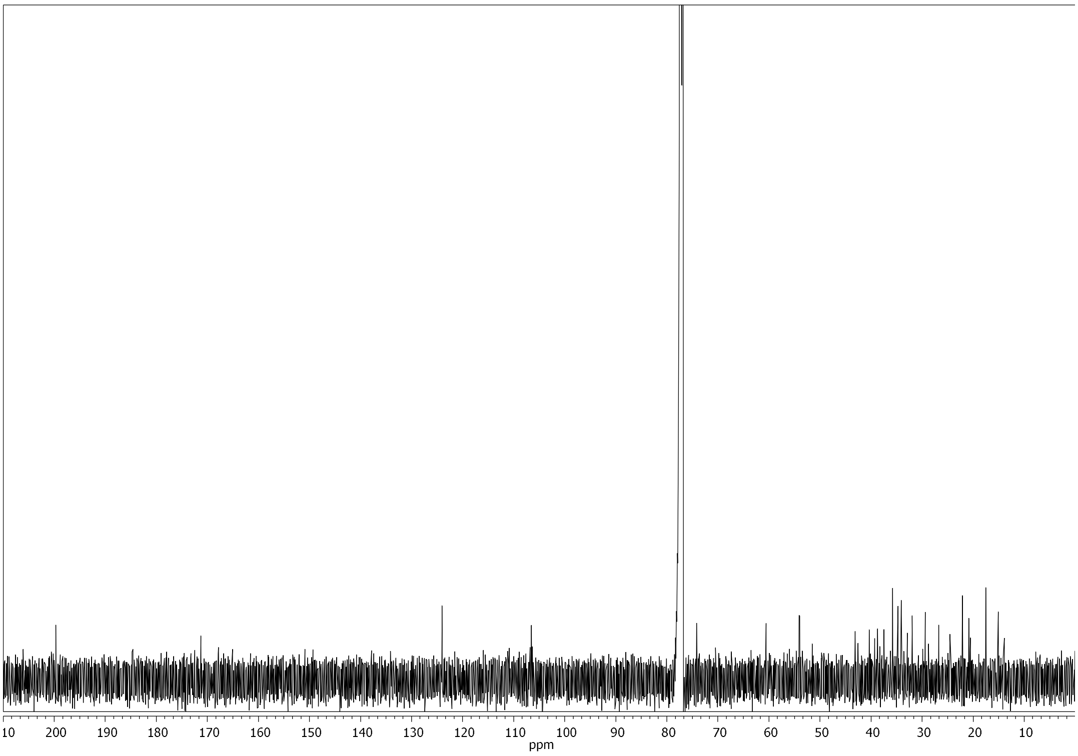

Figure S19. ^{13}C NMR spectrum of compound **3** in $CDCl_3$

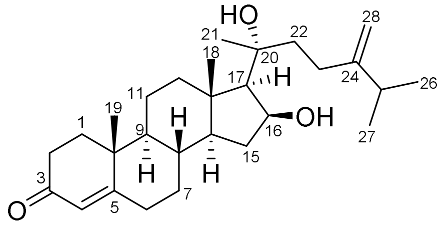

3

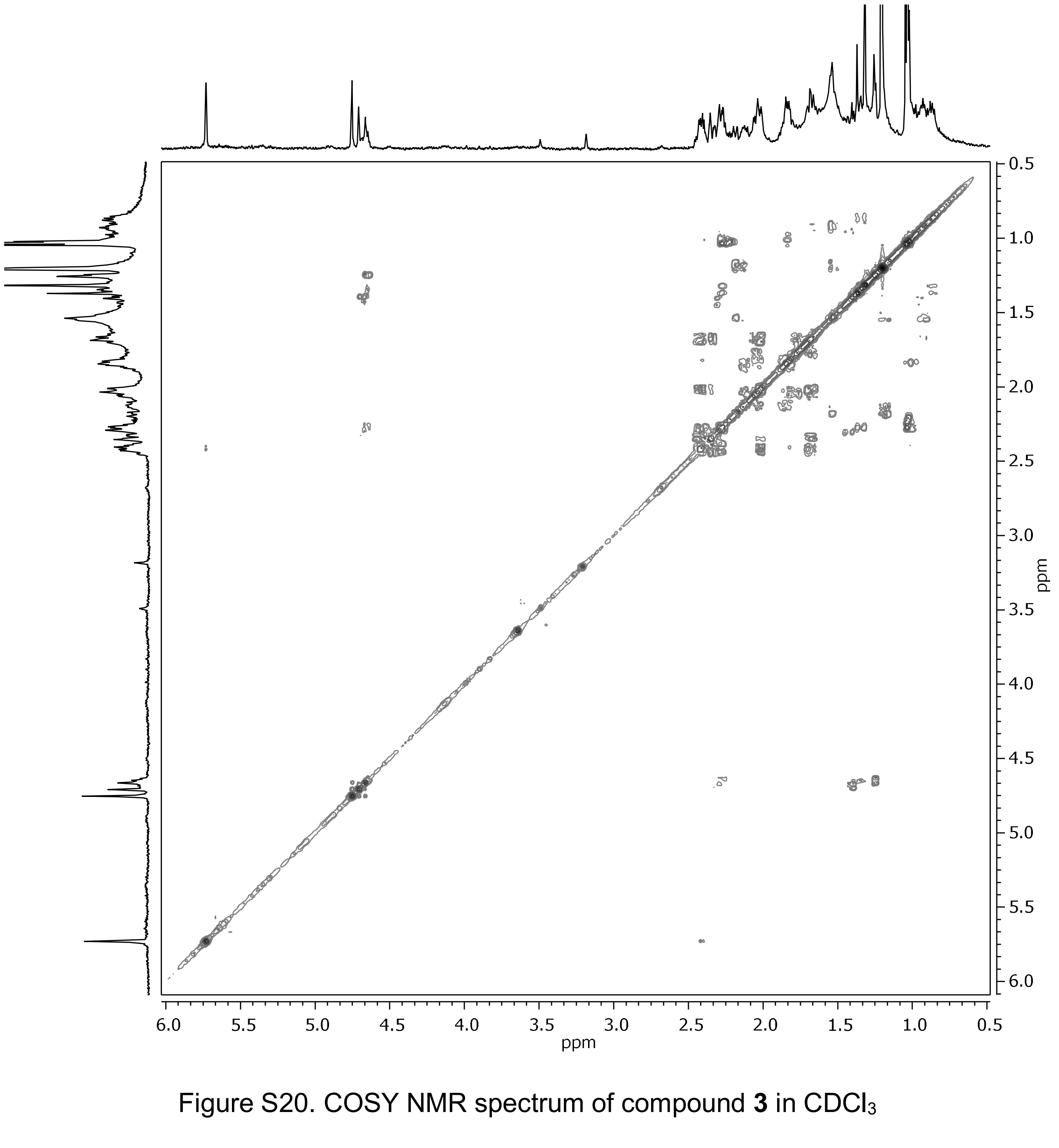

Figure S20. COSY NMR spectrum of compound **3** in $CDCl_3$

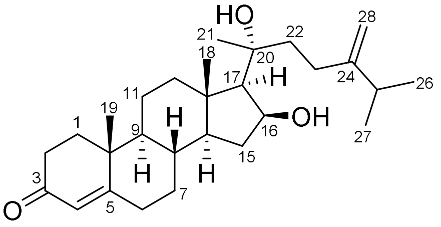

3

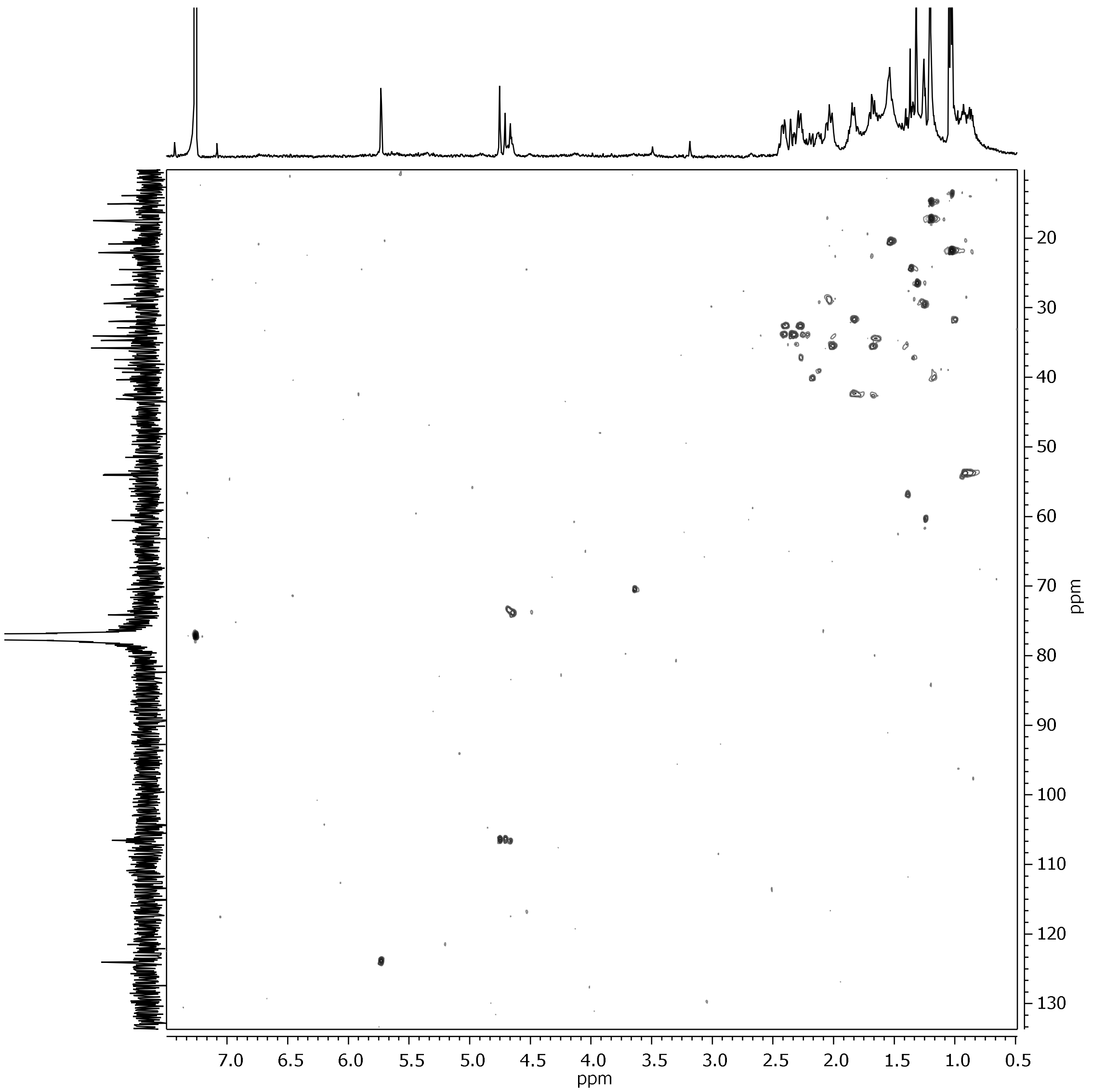

Figure S21. HSQC NMR spectrum of compound **3** in $CDCl_3$

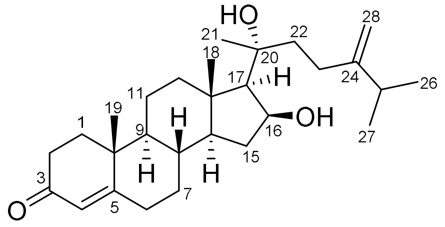

3

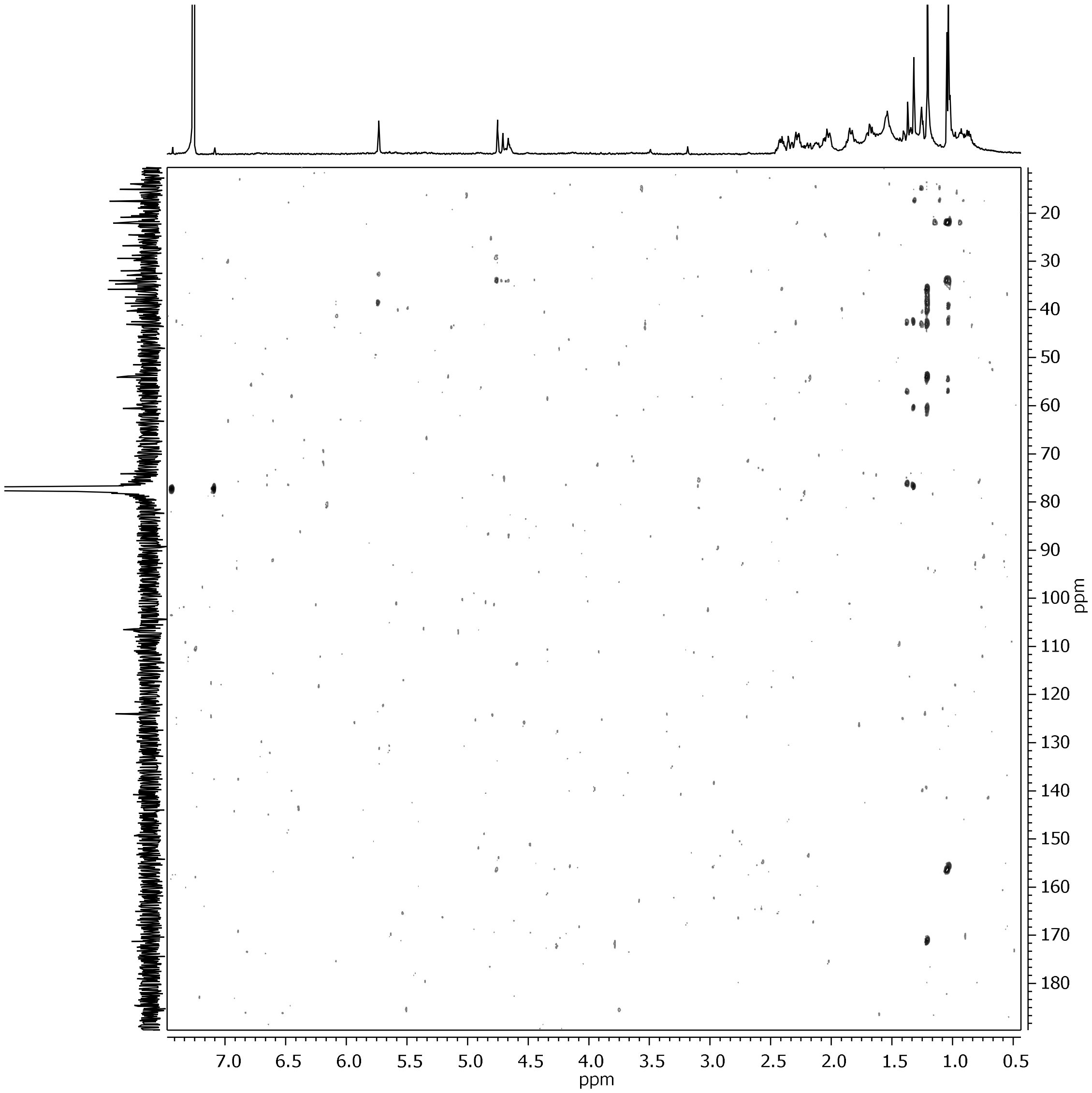

Figure S22. HMBC NMR spectrum of compound **3** in $CDCl_3$

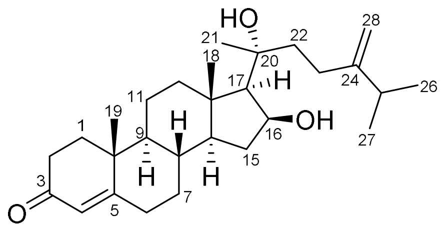

3

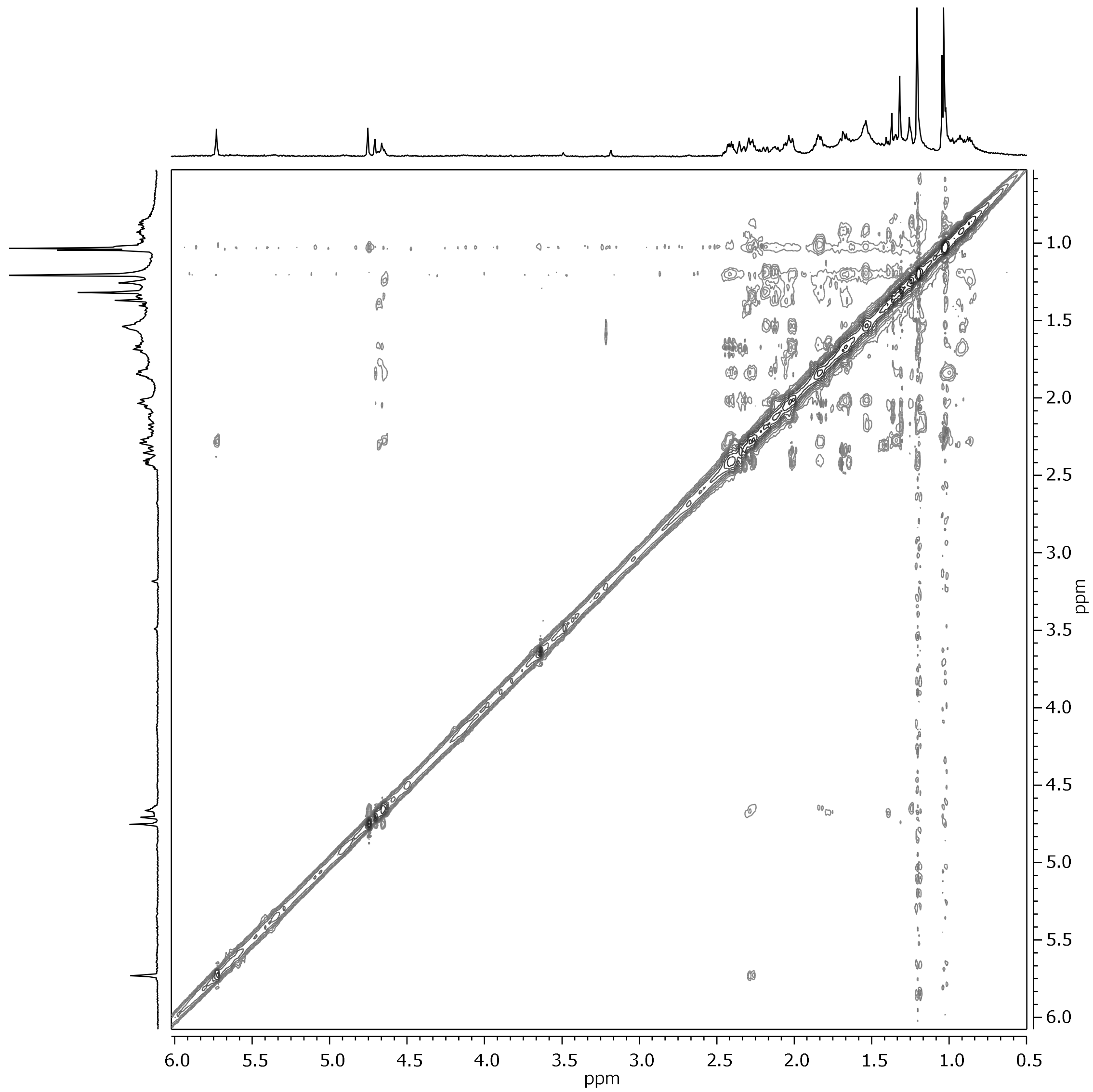

Figure S23. NOESY NMR spectrum of compound **3** in $CDCl_3$

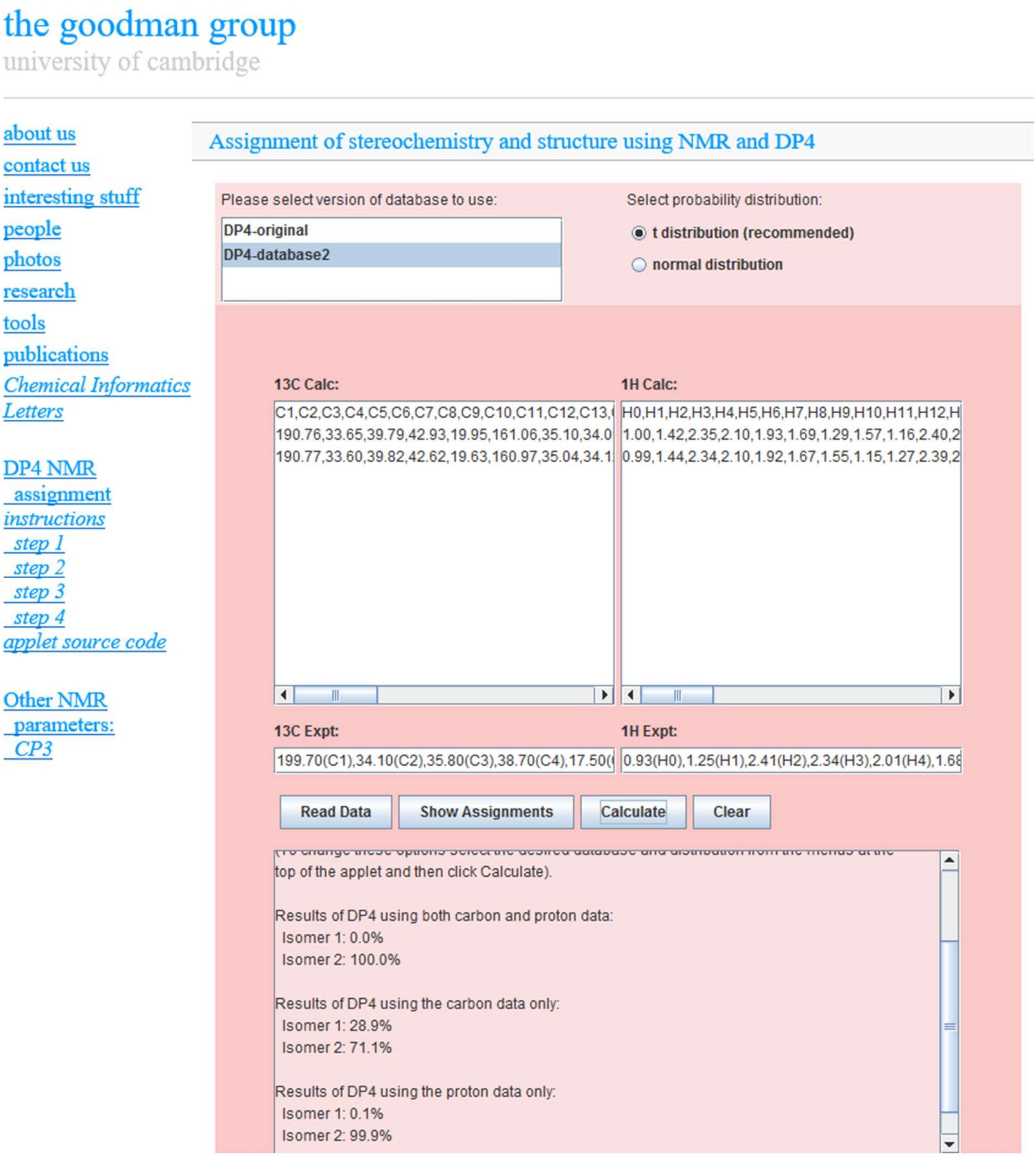

Figure S24. DP4 analysis of compound **3**

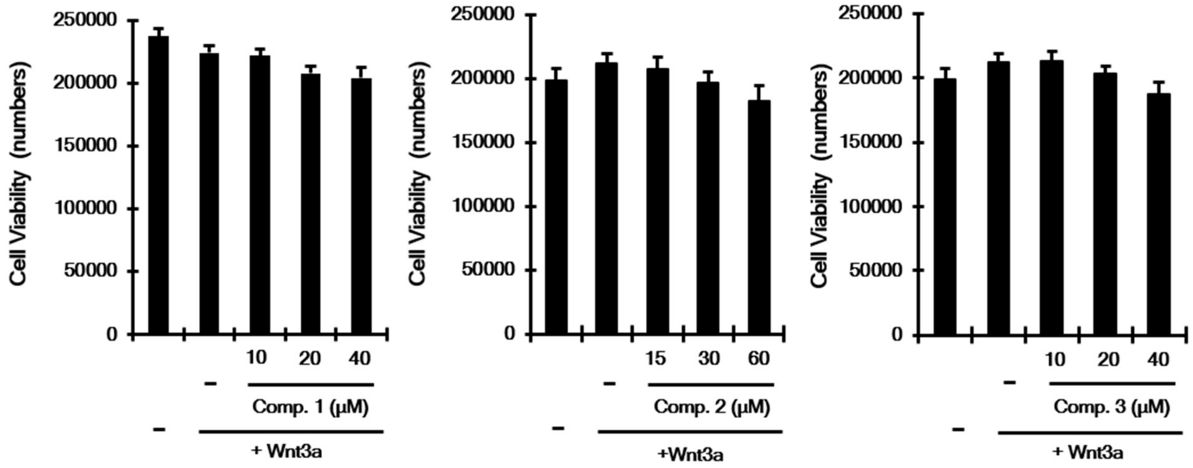

Figure S25. The effect of compounds **1**, **2,** and **3** on the viability of HEK293-FL cells

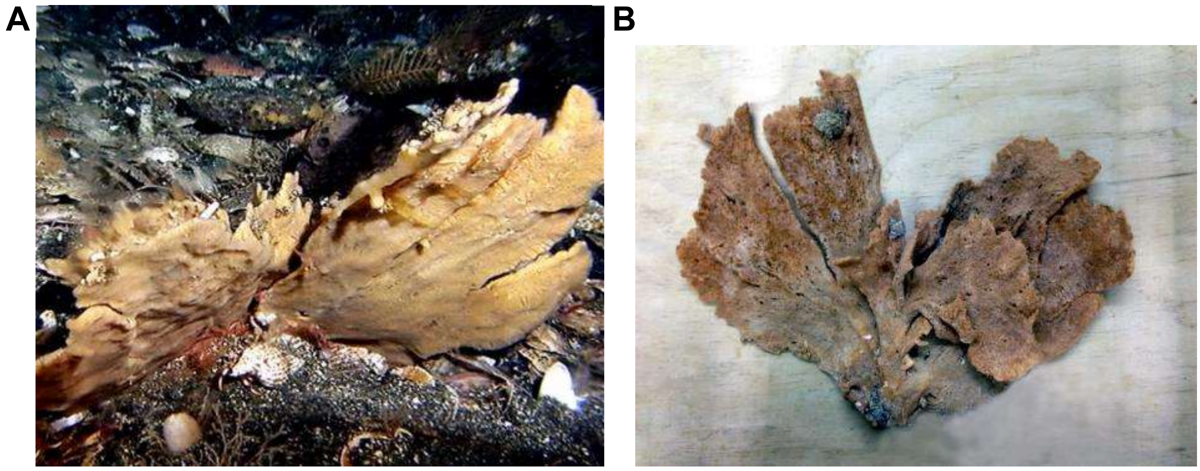

Figure S26. *Monanchora pulchra* Lambe, 1895: (**A**) specimen AB13-0142 in life showing pale orange-yellow colouration, thin surface aquiferous canals and deeply incised margins. (**B**) Specimen AB13-0142, dried upon collection. Figure S26A taken from Stone et al. 2011 (P. 77: 61. *Monanchora pulchra* (Lambe, 1894) Fig. 3).

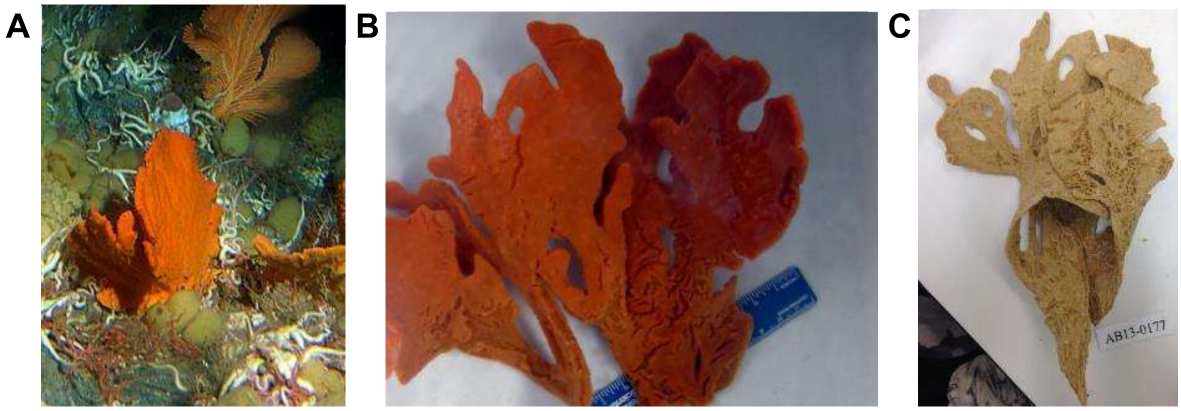

Figure S27. *Monanchora* cf *pulchra* Lambe, 1895: (**A**) specimen AB13–0177 *in situ* at a depth of 80 m showing the deep orange red colouration and marginal oscules in life and the leafy shape. (**B**) Specimen AB13-0177, just after collection showing natural colouration. (**C**) Specimen AB13–0177 (large fragment dried). Figure S27A taken from Stone et al. 2011 (P. 77: 61. *Monanchora pulchra* (Lambe, 1894) Fig. 2)

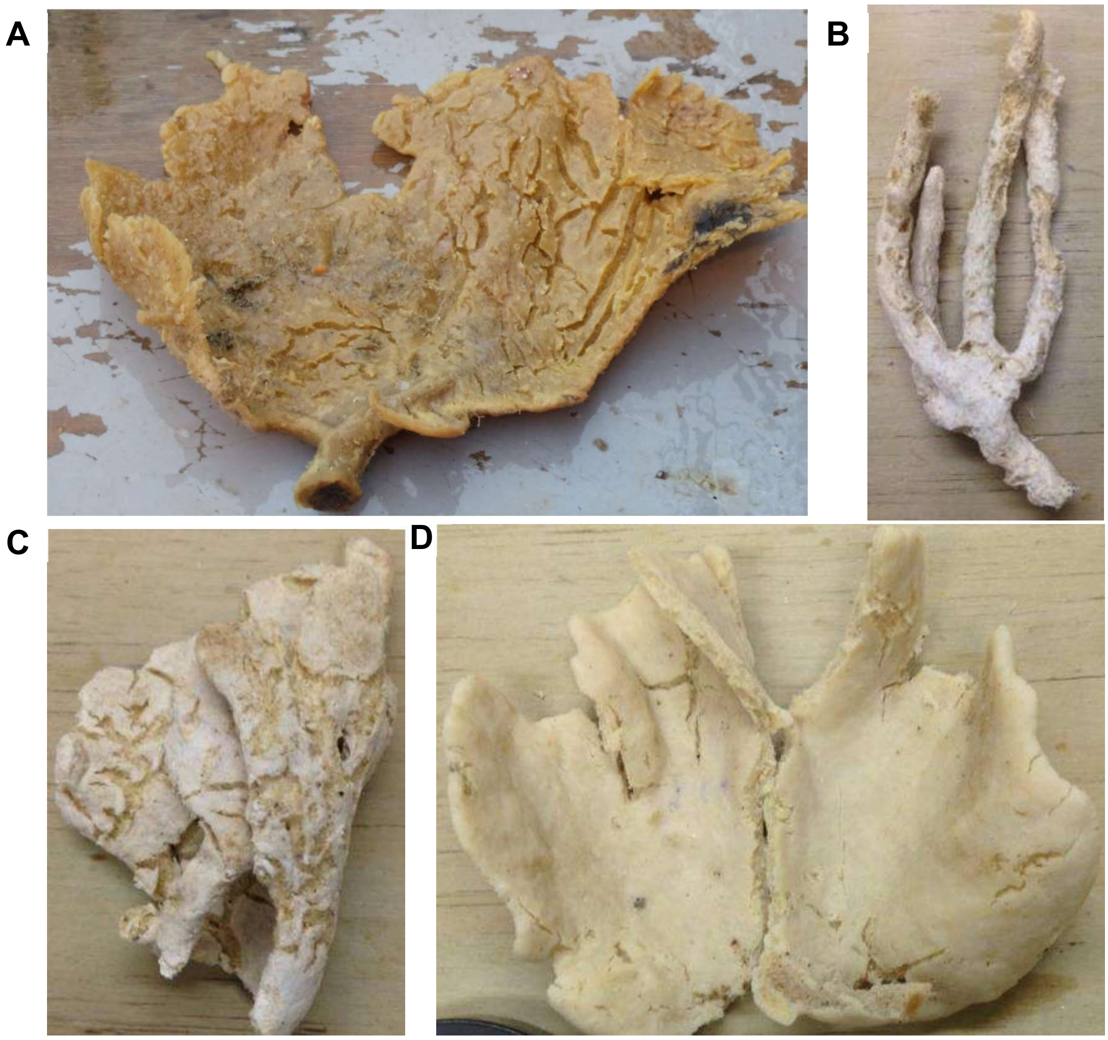

Figure S28. *Monanchora* n. sp. 1 (yellow fan): (**A**) specimen 2010-AK-49 just after collection by trawl, showing the peach colouration and deep surface cracks in life; image courtesy of James Sims, U. Mississippi. (**B**) specimen AB13-0132, dried, with digitate morphology. (**C**) specimen AB13-0247, dried, showing the thick, opaque surface and deep surface cracks in life. (**D**) specimen AB13-0250, ethanol-preserved, showing the thick, opaque surface. All specimens were collected with a bottom trawl in the central Aleutian Islands.

Table S1. Gibbs Free Energy and Boltzmann Population of **1a** for ECD computation

Conformer	Calculated Gibbs Free Energy	Boltzmann Population
1	-1268.009017	41.8%
2	-1268.007495	8.2%
3	-1268.007985	13.8%
4	-1268.008429	22.3%
5	-1268.007264	6.4%
6	-1268.006865	4.2%
7	-1268.006422	2.6%
8	-1268.005273	0.8%

Table S2. The major conformers of diastereomers of compound **2**

Conformers	Boltzmann population(%)	Relative Energy (KJ/mol)
diastereomer a_1	19.994	0
diastereomer a_2	18.273	0.223
diastereomer a_3	17.787	0.29
diastereomer a_4	10.45	1.608
diastereomer a_5	9.153	1.937
diastereomer a_6	9.021	1.973
diastereomer a_7	3.544	4.289
diastereomer a_8	2.385	5.271
diastereomer a_9	1.373	6.64
diastereomer a_10	1.047	7.31
diastereomer a_11	1.044	7.319
diastereomer a_12	1.042	7.324
diastereomer a_13	0.781	8.037
diastereomer a_14	0.764	8.093
diastereomer a_15	0.741	8.168
diastereomer a_16	0.647	8.503
diastereomer a_17	0.601	8.689
diastereomer a_18	0.538	8.962
diastereomer a_19	0.445	9.434
diastereomer a_20	0.371	9.886
diastereomer b_1	22.731	0
diastereomer b_2	16.716	0.762
diastereomer b_3	15.031	1.025
diastereomer b_4	11.939	1.596
diastereomer b_5	9.336	2.206
diastereomer b_6	4.386	4.078
diastereomer b_7	3.624	4.552
diastereomer b_8	3.111	4.93
diastereomer b_9	2.978	5.038
diastereomer b_10	1.246	7.197
diastereomer b_11	1.164	7.366

diastereomer b_12	1.105	7.495
diastereomer b_13	0.953	7.861
diastereomer b_14	0.882	8.053
diastereomer b_15	0.863	8.108
diastereomer b_16	0.824	8.224
diastereomer b_17	0.6	9.01
diastereomer b_18	0.564	9.161
diastereomer b_19	0.543	9.255
diastereomer b_20	0.53	9.317
diastereomer b_21	0.471	9.612
diastereomer b_22	0.403	9.996

Table S3. Experimental and calculated NMR chemical shift values (ppm) of compound **2** with diastereomers (diastereomers a and b are described in the main text)

Number	Experimental	Diastereomer a	Diastereomer b
C-1	35.8	39.88	39.78
C-2	33.1	33.58	33.65
C-3	199.8	190.63	190.65
C-4	124.0	123.44	123.45
C-5	171.6	161.22	161.19
C-6	34.1	35.15	35.22
C-7	32.1	34.09	33.98
C-8	35.9	39.06	39.08
C-9	54.2	56.54	56.54
C-10	38.8	42.73	42.58
C-11	21.1	24.23	24.22
C-12	38.5	40.15	40.28
C-13	44.0	47.09	46.80
C-14	55.5	56.61	56.69
C-15	24.4	26.77	26.59
C-16	24.8	27.81	28.21
C-17	59.1	61.24	60.73
C-18	13.4	15.55	15.79
C-19	17.6	19.84	19.75
C-20	138.5	134.43	134.79
C-21	17.8	21.01	21.11
C-22	128.1	128.44	127.01
C-23	73.7	73.21	72.86
C-24	34.7	36.30	36.39
C-25	18.5	20.30	20.15
C-26	18.3	19.49	19.26
H-1a	2.02	1.91	1.90
H-1b	1.67	1.71	1.69
H-2a	2.40	2.34	2.34
H-2b	2.28	2.11	2.10
H-4	5.73	5.87	5.87
H-6a	2.43	2.37	2.37
H-6b	2.36	2.16	2.15
H-7a	1.84	1.77	1.77
H-7b	1.03	1.05	1.05
H-8	1.52	1.67	1.68
H-9	0.94	1.06	1.05
H-11a	1.58	1.57	1.55
H-11b	1.42	1.53	1.52
H-12a	1.84	1.82	1.79
H-12b	1.17	1.21	1.22
H-14	1.08	1.28	1.29
H-15a	1.73	1.72	1.70
H-15b	1.23	1.39	1.39
H-16a	1.84	2.01	1.99
H-16b	1.84	1.75	1.74

H-17	2.08	2.19	2.11
H-18	0.63	0.69	0.61
	0.63	0.73	0.80
	0.63	1.02	1.16
H-19	1.18	1.53	1.53
	1.18	1.29	1.11
	1.18	1.11	1.28
H-21	1.70	1.48	1.71
	1.70	1.95	1.63
	1.70	1.48	1.49
H-22	5.24	5.68	5.68
H-23	4.15	4.41	4.44
H-24	1.70	1.83	1.90
H-25	0.96	1.43	1.33
	0.96	0.78	0.81
	0.96	0.82	0.83
H-26	0.88	0.77	0.94
	0.88	1.27	0.78
	0.88	0.75	1.17

Table S4. The major conformers of diastereomers of compound **3**

Conformers	Boltzmann population(%)	Relative Energy (KJ/mol)
diastereomer a_1	10.73	0
diastereomer a_2	10.58	0.036
diastereomer a_3	8.90	0.465
diastereomer a_4	7.40	0.92
diastereomer a_5	7.12	1.018
diastereomer a_6	4.81	1.99
diastereomer a_7	4.71	2.045
diastereomer a_8	4.49	2.16
diastereomer a_9	4.47	2.172
diastereomer a_10	4.37	2.226
diastereomer a_11	3.91	2.504
diastereomer a_12	2.73	3.394
diastereomer a_13	2.64	3.477
diastereomer a_14	2.50	3.608
diastereomer a_15	2.25	3.869
diastereomer a_16	2.23	3.898
diastereomer a_17	1.56	4.785
diastereomer a_18	1.45	4.963
diastereomer a_19	1.44	4.978
diastereomer a_20	1.38	5.08
diastereomer a_21	1.38	5.083
diastereomer a_22	1.28	5.264
diastereomer a_23	1.17	5.5
diastereomer a_24	1.03	5.81
diastereomer a_25	0.97	5.971
diastereomer a_26	0.78	6.496
diastereomer a_27	0.63	7.029
diastereomer a_28	0.55	7.383
diastereomer a_29	0.47	7.761
diastereomer a_30	0.44	7.934
diastereomer a_31	0.33	8.607

diastereomer a_32	0.32	8.691
diastereomer a_33	0.32	8.749
diastereomer a_34	0.24	9.475
diastereomer a_35	0.23	9.492
diastereomer a_36	0.20	9.897
diastereomer b_1	13.98	0
diastereomer b_2	11.17	0.557
diastereomer b_3	9.86	0.866
diastereomer b_4	8.35	1.277
diastereomer b_5	7.70	1.479
diastereomer b_6	6.72	1.817
diastereomer b_7	6.40	1.938
diastereomer b_8	5.40	2.357
diastereomer b_9	4.58	2.766
diastereomer b_10	4.29	2.928
diastereomer b_11	4.14	3.016
diastereomer b_12	2.44	4.331
diastereomer b_13	2.34	4.435
diastereomer b_14	2.30	4.477
diastereomer b_15	2.19	4.591
diastereomer b_16	1.37	5.762
diastereomer b_17	1.33	5.836
diastereomer b_18	1.14	6.219
diastereomer b_19	0.89	6.835
diastereomer b_20	0.88	6.864
diastereomer b_21	0.78	7.168
diastereomer b_22	0.66	7.558
diastereomer b_23	0.43	8.653
diastereomer b_24	0.41	8.728
diastereomer b_25	0.28	9.675

Table S5. Experimental and calculated NMR chemical shift values (ppm) of compound **3** with diastereomers (diastereomers a and b are described in the main text)

Number	Experimental	Diastereomer a	Diastereomer b
C-3	199.70	190.76	190.77
C-2	34.10	33.65	33.60
C-1	35.80	39.79	39.82
C-10	38.70	42.93	42.62
C-19	17.50	19.95	191.91
C-5	171.10	161.06	160.97
C-6	32.90	35.10	35.04
C-7	32.00	34.09	34.12
C-8	34.70	37.85	37.97
C-9	54.00	56.16	56.17
H-9	0.93	1.00	0.99
C-11	20.90	23.93	23.94
C-12	40.40	41.53	41.07
C-13	43.10	47.68	47.86
C-14	53.90	54.55	54.60
C-15	37.50	39.75	39.85
C-16	74.20	75.12	76.10
C-17	60.60	59.49	59.46
H-17	1.25	1.42	1.44
C-20	76.50	76.43	76.45
C-21	26.70	27.96	30.25
C-22	42.60	46.96	42.30
C-23	28.70	39.74	38.24
C-24	156.30	155.19	154.96
C-25	33.90	34.87	35.15
C-26	22.10	25.92	24.82
C-27	22.10	25.02	25.95
C-28	106.50	105.74	106.35
C-4	124.10	123.33	123.40
H-2a	2.41	2.35	2.34
H-2b	2.34	2.10	2.10
H-1a	2.01	1.93	1.92
H-1b	1.68	1.69	1.67
		1.29	1.55
H-19	1.21	1.57	1.15
		1.16	1.27
H-6a	2.40	2.40	2.39
H-6b	2.27	2.14	2.14
H-7a	1.83	1.69	1.67
H-7b	1.00	1.01	1.00
H-11a	1.52	1.47	1.70
H-11b	1.52	1.72	1.46
H-12a	2.17	2.21	2.21
H-12b	1.17	1.10	1.19
H-15a	2.27	2.11	2.11
H-15b	1.34	1.41	1.39

		1.04	1.41
H-21	1.32	1.60	1.14
		1.22	1.51
H-22a	1.68	2.10	1.85
H-22b	1.68	1.70	1.77
H-23a	2.03	2.16	2.23
H-23b	2.03	1.99	2.11
H-25	2.23	2.33	2.37
		1.01	1.06
H-26	1.03	1.18	1.10
		1.06	1.09
		1.19	1.06
H-27	1.03	1.03	1.17
		1.04	1.04
H-28a	4.75	4.99	5.00
H-28b	4.68	4.97	4.98
H-4	5.74	5.85	5.83
H-8	1.65	1.88	1.87
H-14	0.87	0.96	0.95
C-18	15.10	17.83	17.54
	1.21	1.35	1.41
H-18	1.21	1.55	1.57
	1.21	1.56	1.33
H-16	4.65	4.67	4.71
